# Synthesis of Nitrogen-Containing Heterocyclic Scaffolds through Sequential Reactions of Aminoalkynes with Carbonyls

**DOI:** 10.3390/molecules28124725

**Published:** 2023-06-12

**Authors:** Antonio Arcadi, Valerio Morlacci, Laura Palombi

**Affiliations:** Dipartimento di Scienze Fisiche e Chimiche, Università degli Studi dell’Aquila, Via Vetoio, 67100 Coppito, L’Aquila, Italy; valerio.morlacci@graduate.univaq.it (V.M.); laura.palombi@univaq.it (L.P.)

**Keywords:** sequential reactions, aminoalkynes, heterocycles, metal catalysis

## Abstract

Sequential reactions of aminoalkynes represent a powerful tool to easily assembly biologically important polyfunctionalized nitrogen heterocyclic scaffolds. Metal catalysis often plays a key role in terms of selectivity, efficiency, atom economy, and green chemistry of these sequential approaches. This review examines the existing literature on the applications of reactions of aminoalkynes with carbonyls, which are emerging for their synthetic potential. Aspects concerning the features of the starting reagents, the catalytic systems, alternative reaction conditions, pathways and possible intermediates are provided.

## 1. Introduction

Aminoalkynes are bifunctional derivatives that can undergo a diverse array of transformations. They offer sequential reactions with an electrophile and a nucleophile, and are ideal for cascade reactions. Sequential reactions represent a powerful tool to build up simple or more complex polyfunctionalized organic scaffolds from readily available reagents with high efficiency, selectivity, and atom economy [1,2,3]. Recently, applications of sequential reactions of aminoalkynes have been a very active research field in organic synthesis and medicinal chemistry. In particular, sequential reactions of β-, γ-, and δ-aminoalkynes to afford a variety of heterocyclic scaffolds were explored. Inactivated alkynes moieties are not very reactive toward nucleophiles. Their behavior changes by activation of the C-C triple bond by a metal catalyst. Various biologically important nitrogen heterocycles were directly synthesized in an easy way by means of intramolecular hydroamination of aminoalkynes in the presence of several transition metal as well as lanthanide catalysts [4,5]. The aptitude to form π- and σ-complexes can help in the choice of catalysts for the desired transformations when bi- or polyfunctional substrates are involved [6]. The reaction of γ- and δ-aminoalkynes with sulfonyl azides in the presence of Ru_3_(CO)_12_ catalyst efficiently afforded cyclic amidines of relevance in medicinal and coordination chemistry as well as in materials science [7]. The gold(I)-catalyzed tandem cyclization of γ-aminoalkynes with alkynes in water led to diversely substituted pyrrolo[1,2-*a*]quinolines [8]. Zhou et al. extended this reaction using less active terminal amidoalkynes in similar conditions [9]. The CuCl-catalyzed cascade transformation of internal β-aminoalkynes with alkynes under microwave irradiation gave diversely substituted tetrahydropyrrolo[1,2-*a*]quinolones [10]. An intramolecular gold-catalyzed hydroamination/aza-Diels–Alder tandem process of β-/γ-aminoalkynes with high regio- and diastereoselectivity and up to almost complete chemoselectivity showed great efficiency in a one-pot approach to the complex nitrogen heterocyclic derivatives of medicinal importance, such as the one-step synthesis of incargranine B aglycone and (±)-seneciobipyrrolidine (I) [11]. Faňanás, Rodríguez, and co-workers [12] described the preparation of complex pyrrolidines from readily available *N*-Boc-derived β-aminoalkynes and alkenes or alkynes through relay actions of Pt^II^ or Brønsted acids [13]. The reaction of α-aminoalkynes with carbon monoxide and selenium yielded 5-alkylideneselenazolin-2-ones stereoselectively via cycloaddition of in situ generated carbamoselenoates to a carbon–carbon triple bond. β-Aminoalkyne also afforded the corresponding six-membered selenium-containing heterocycle with the aid of CuI [14]. An operationally simple palladium-catalyzed intramolecular hydroaminocarbonylation of a variety of aminoalkynes directly provided a viable approach to a variety of valuable seven- and eight-membered lactams with high chemoselectivity and regioselectivity [15]. A sequential heterogeneous PtI_2_-catalyzed hydration of δ-aminoalkynes followed by intramolecular cyclization and intermolecular addition as well as ring-expansion cascade reaction with another electron-deficient alkynes was developed for the synthesis of various eight-membered nitrogen heterocycles with excellent yields under mild reaction conditions. The simple PtI_2_ could be easily recycled [16]. Moreover, a PtI_2_-catalyzed formal three-component cascade cycloaddition reactions between γ-aminoalkynes and electron-deficient alkynes gave highly functionalized cyclohexadiene-*b*-pyrrolidines with good yields [17]. Finally, among the domino and multicomponent processes that involve aminoalkynes, their cascade reactions with carbonyl derivatives stand out as a highly versatile tool to build up libraries of nitrogen-containing heterocyclic scaffolds with diversity and molecular complexity. This review will examine the literature on this last topic and is organized according to the structure of the aminoalkyne substrate. Aspects concerning the features of the catalytic systems, the substrate scope, insight into the reaction pathways, possible intermediates, and alternative conditions are discussed.

## 2. Sequential Reactions of α-Aminoalkynes (Propargylamines) with Carbonyls

**Scheme 1:**
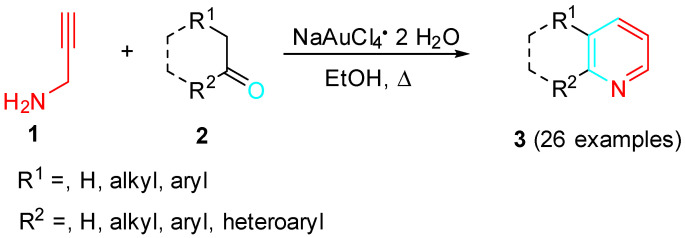
Gold-catalyzed synthesis of pyridines from propargylamine and carbonyls.

Propargylic amine derivatives represent useful α-aminoalkynes building blocks for the construction of nitrogen-containing heterocyclic scaffolds through their sequential reactions with carbonyls. The gold-catalyzed reaction of propargylamine **1** with dialkyl acyclic/cyclic ketones, methyl, aryl/heteroaryl ketones and aldehydes bearing α-hydrogens **2** allowed a simple approach to pyridines **3** through a sequential amination–cyclization–aromatization cascade (Scheme 1) [18].

**Scheme 2:**
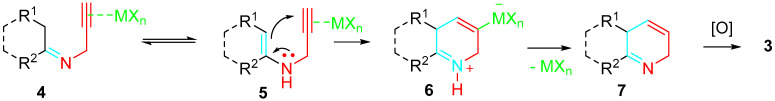
Transition metal-catalyzed sequential amination–cyclization–aromatization reaction.

The catalyst was envisaged to promote both the amination of carbonyl compounds **2** and the regioselective 6*-endo-*dig cyclization of the *N*-propargylenamine (*N*-propargyldienamine) intermediate **5** (Scheme 2).

**Scheme 3:**
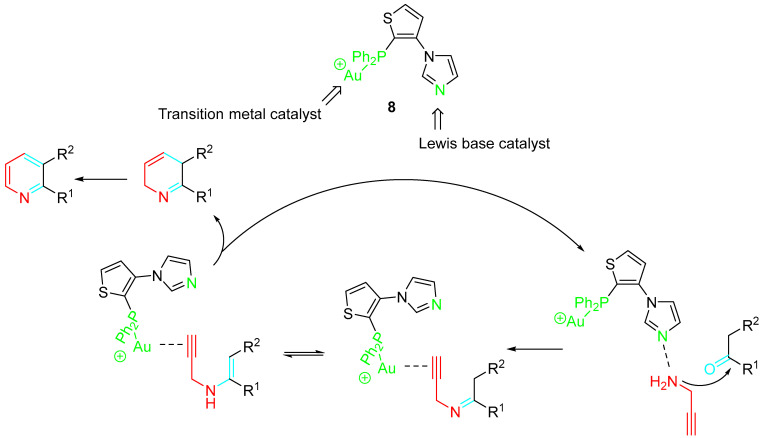
Sequential reactions of carbonyl compounds and propargylamine catalyzed by Au-complex **8**.

A variety of catalysts were tested in the reaction of **1** with **2**. In particular, NaAuCl_4_·2H_2_O resulted in a highly efficient catalyst. Moreover, Au **8** was synthesized and applied as bifunctional catalyst. It was found that imidazolyl group acted as a Lewis base to catalyze the condensation of carbonyl compounds with propargylamine to form the imino intermediate, and the involved Au^+^-complex species activated the alkynyl moiety to give the dehydropyridine derivative, which underwent auto-oxidation reaction to afford the target pyridines (Scheme 3) [19].

**Scheme 4:**
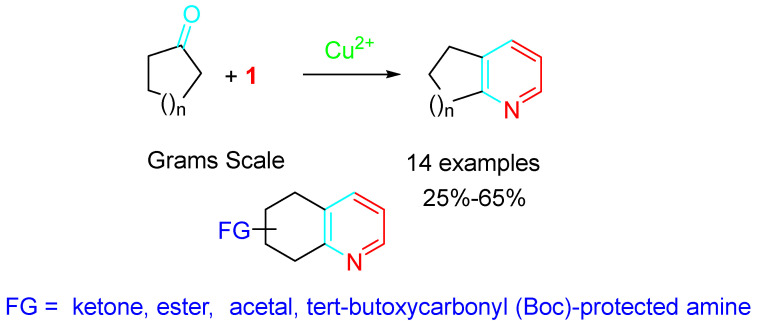
Cu-catalyzed pyridine synthesis from cyclic ketones and propargylamine.

Copper salts were also effective catalysts in the reaction of cyclic ketones with propargylamine, and the highest product yields were observed in isopropanol (*i*-PrOH) in the presence of 5.0 mol% CuCl_2_ in air. Decreased yields among cyclic ketones were observed in the following order: six-membered >> eight-membered > five-membered ∼ seven-membered. However, the inexpensiveness of the catalyst and the tolerance to a wide number of functional groups (FG) in the ketone make the procedure very suitable for large-scale preparation of fused pyridines (Scheme 4) [20].

**Scheme 5:**
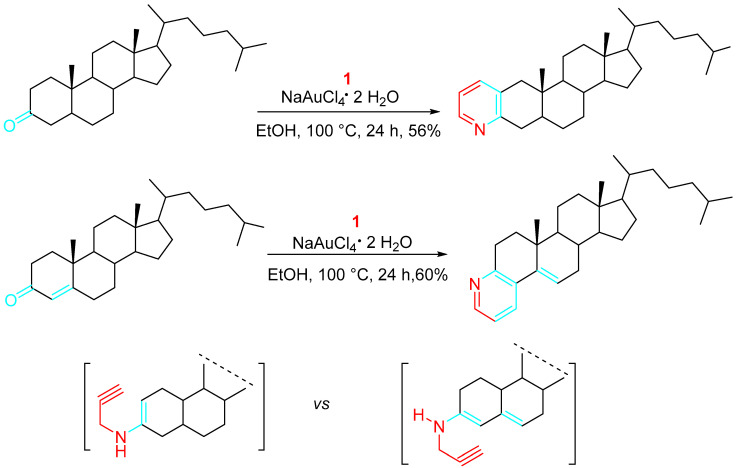
Linear vs. angular steroidal pyridines.

Selective aspects of the reaction of steroidal carbonyls with propargylamine were investigated. According to the results, the regioselective pyridine fusion to the cyclic skeleton was addressed by suitable choice between the substrate bearing a saturated or conjugated carbonyl group (Scheme 5) [18].

**Scheme 6:**
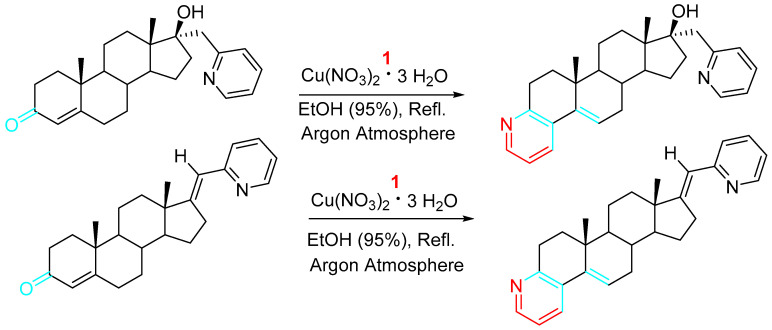
Copper-catalyzed synthesis of A-ring fused pyridine D-modified androstane derivatives.

Analogously, new A-ring pyridine fused androstanes in 17a-homo-17-oxa (D-homo lactone), 17α-picolyl or 17(*E*)-picolinylidene series were obtained by reacting 4-en-3-one or 4-ene-3,6-dione D-modified androstane derivatives with propargylamine under the presence of a Cu(II) catalyst, and evaluated for potential anticancer activity in vitro (Scheme 6) [21].

**Scheme 7:**
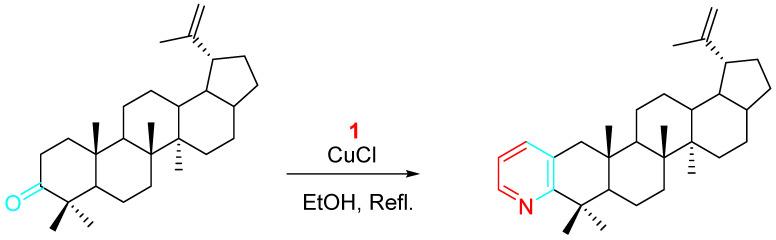
Copper-catalyzed synthesis of betulin derivatives.

Similarly, the efficient synthesis of pyridine rings fused to the 3,4-positions of the steroid nucleus was described via the Cu(II)-catalyzed reaction of propargylamine with 17β-hydroxyandrost-4-en-3-one, 17α-methyl-17β-hydroxyandrost-4-en-3-one, or 17β-hydroxyestr-4-en-3-one [22]. The procedure was also applied to the synthesis of heterocyclic betulin derivatives (Scheme 7) [23,24].

**Scheme 8:**
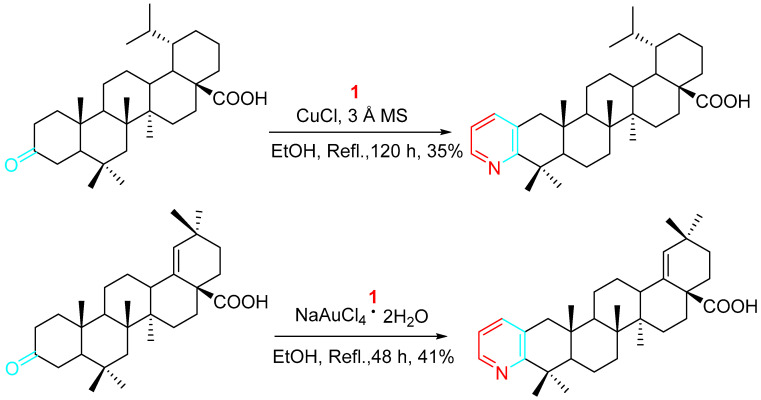
Optimization of the synthesis of relevant steroidal pyridines.

Optimization of the synthesis of steroidal pyridines was tried by prolonging the reaction time and varying the catalyst loading. In some cases, the use of NaAuCl_4_·2H_2_O instead of CuCl and the addition of activated molecular sieves (MS) to the reaction mixture led to significant improvement (Scheme 8) [25].

**Scheme 9:**
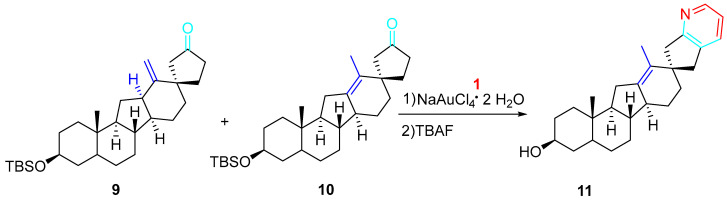
Gold-catalyzed synthesis of the carbacycloamine analogue **11**.

Several other applications of the methodology accomplished the preparation of significant scaffolds. Indeed, the chemical synthesis of highly potent and acid-stable inhibitors of hedgehog signaling carbacyclopamine analogue **11** was reported. The gold-catalyzed amination–annulation–aromatization sequence applied to the inseparable mixture of the isomers **9** and **10** regioselectively furnished, after removal of the *tert*-butyldimethylsilyl ether (tetrabutylammonium fluoride, THF, 25 °C), carbacyclopamine analogue **11** with 36% overall yield for the two steps (Scheme 9) [26].

**Scheme 10:**
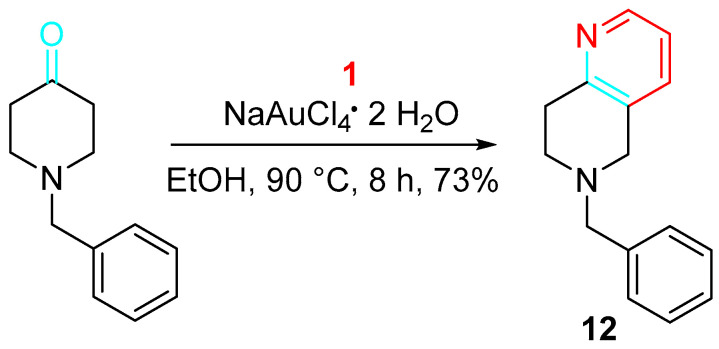
Gold-catalyzed synthesis of the potassium channel modulator **12**.

Furthermore, the gold-catalyzed reaction of 1-benzylpiperidin-4-one with propargylamine efficiently afforded the potassium channel modulator 6-benzyl-5,6,7,8-tetrahydro-1,6-naphthirine **12** (Scheme 10) [27].

**Scheme 11:**
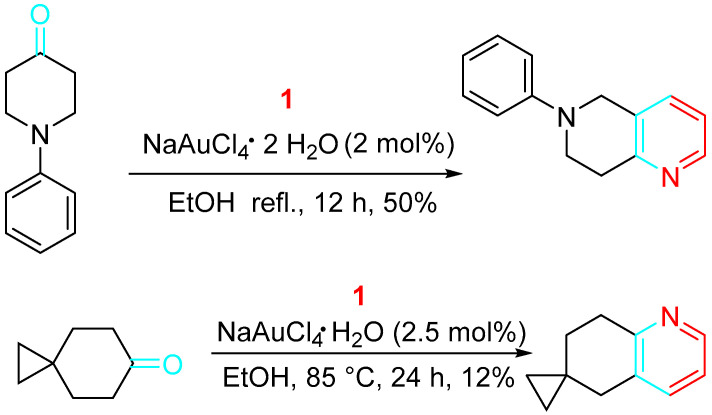
Synthesis of Wnt signal path inhibitors.

The methodology was extended to the synthesis of Wnt signal path inhibitors (Scheme 11) [28,29].

**Scheme 12:**
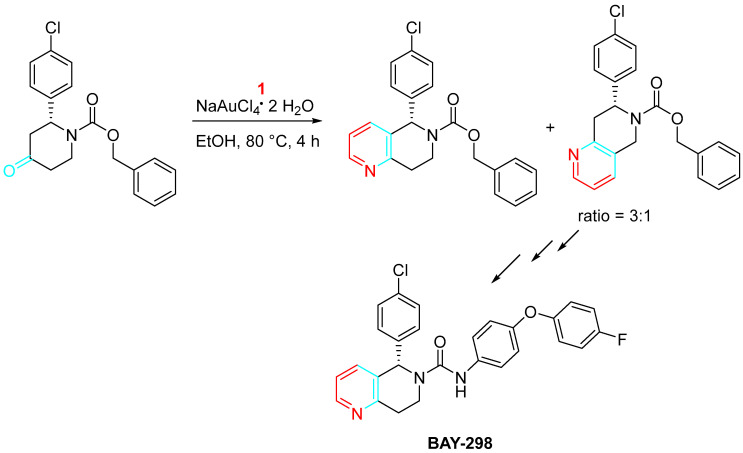
Gold-catalyzed synthesis of BAY-298.

The gold-catalyzed synthesis of BAY-298, a nanomolar small molecule (SMOL) hLH-R antagonist, reducing sex hormone levels in vivo has been described (Scheme 12) [30].

**Scheme 13:**
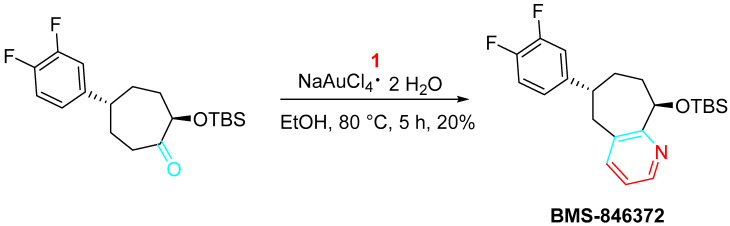
Synthesis of BMS-846372.

Moreover, the gold-catalyzed sequential condensation–cyclization–aromatization procedure was extended as the key step for the preparation of BMS-846372, a potent and orally active human CGRP receptor antagonist employed for migraine therapy (Scheme 13) [31].

**Scheme 14:**
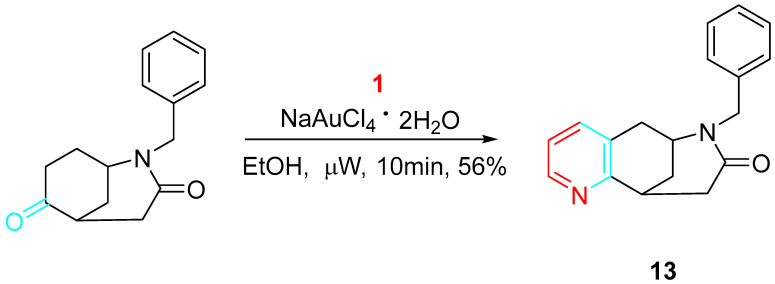
Gold-catalyzed synthesis of the benzomorphane derivative **13**.

The methodology also resulted in a viable tool for the preparation of aryl and heteroaryl derivatives of benzomorphanes **13**, pharmacologically active as inhibitors of 11ß-hydroxysteroid dehydrogenase (HSD1) (Scheme 14) [32].

**Scheme 15:**
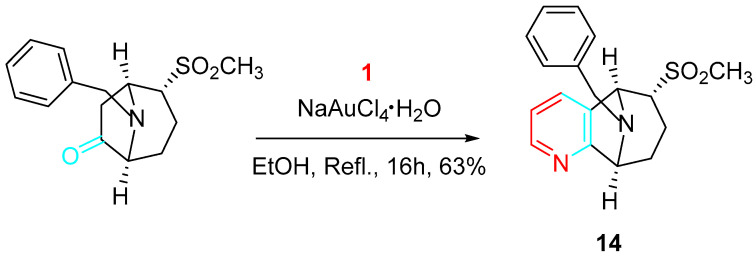
Gold-catalyzed synthesis of the tropane-related scaffold **14**.

The synthetic approach has yielded a number of scaffolds suitable for the design of performance-diverse screening libraries (Scheme 15) [33].

**Scheme 16:**
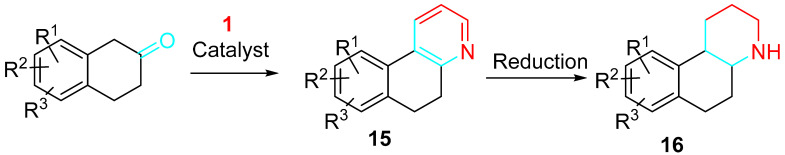
Synthesis of 11β-hydroxysteroid dehydrogenase inhibitors.

The reaction of 2-tetralones and propargylamine in the presence of complexes of gold or copper, preferably NaAuCl_4_ and CuC1, was employed to synthesize octahydrobenzoquinoline derivatives **16** as inhibitors of 11β-hydroxysteroid dehydrogenase for the treatment of metabolic disorders, such as metabolic syndrome, diabetes, obesity, and dyslipidemia. The reaction is usually run in alcohols at temperatures ranging from 20 to 120 °C through conventional heating or microwave irradiation. The resulting pyridine was reduced to the corresponding piperidine (Scheme 16) [34].

**Scheme 17:**
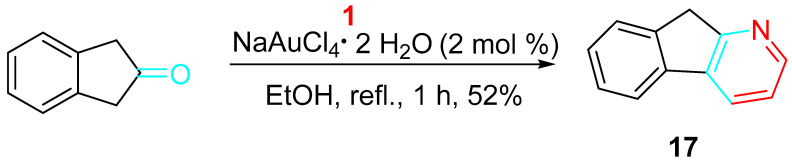
Gold-catalyzed synthesis of the ligand **17**.

The sequential gold-catalyzed condensation/annulation reaction of the 1,3-dihyrdo-*2H*-inden-2-one with the propargylamine provided the corresponding *9H*-indeno [2,1-*b*]pyridine **17** as the ligand for the synthesis of an olefin polymerization catalyst (Scheme 17) [35].

**Scheme 18:**
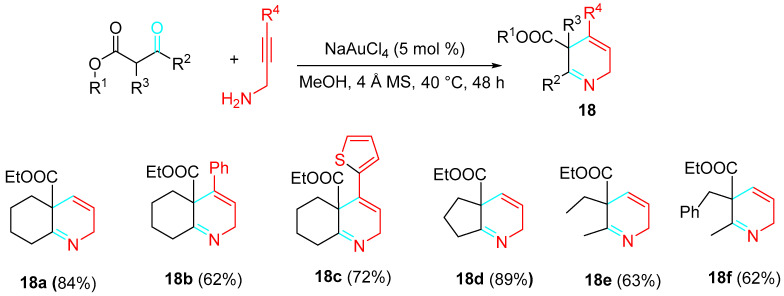
Gold-catalyzed synthesis of 2,5-dihydropyridines **18**.

The gold(III)-catalyzed reaction of simple β-ketoesters with propargylamines achieved the synthesis of potentially bioactive 2,5-dihydropyridines **18** with satisfactory yields. The best results were observed using 5 mol% of the cheaper NaAuCl_4_ in MeOH as solvent. The dichloro(2-pyridinecarboxilato)gold (pic)AuCl_2_) resulted in a less effective catalyst, and the reaction failed to occur in the presence of (Ph_3_P)AuCl/AgOTf catalytic system or by using platinum(II) and platinum(IV) catalysts. Recovery of the starting materials when triflic acid (TfOH) was used instead of NaAuCl_4_ ruled out the formation of the product by Brønsted acid catalysis. Propargylamines unsubstituted at the triple bond (R^4^=H) or with an aromatic ring at this position gave higher yields than propargylamines bearing an aliphatic chain at the same position (Scheme 18) [36].

**Scheme 19:**
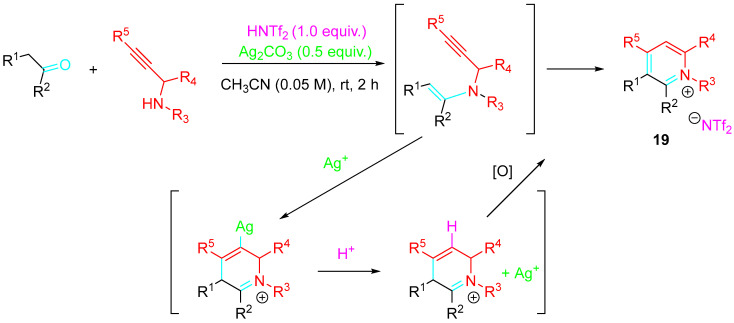
Synthesis of pyridinium salts.

Substituted pyridinium salts **19** were obtained under mild conditions by a condensation reaction between carbonyls and propargylamine under the presence of an Ag_2_CO_3_/HNTf_2_ synergistically acting catalyst system. The one-pot transformation should proceed via sequential 6-*endo*-dig cyclization of the in situ generated propargylenamine/protonolysis of the resulting vinyl–silver intermediate. The silver(I)-catalyzed cyclization reaction was exclusively selective for the formation of six-membered rings. Only 6-*endo*-dig cyclized pyridinium products were obtained, even with substrates bearing an electron-withdrawing group at the acetylenic position, which underwent unusual inversion of the reactivity usually observed in Michael-type reactions. CH_3_CN was the solvent of choice in this one-pot transformation (Scheme 19) [37].

**Scheme 20:**
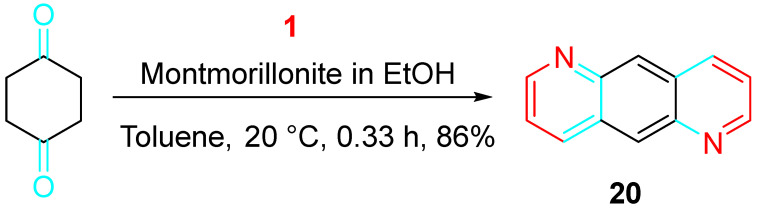
Metal-free synthesis of the hetero-anthracene derivative **20**.

Interestingly, hetero-anthracene derivatives such as **20**, used in the preparation of organic light-emitting devices, were practically obtained under metal-free conditions (Scheme 20) [38].

**Scheme 21:**
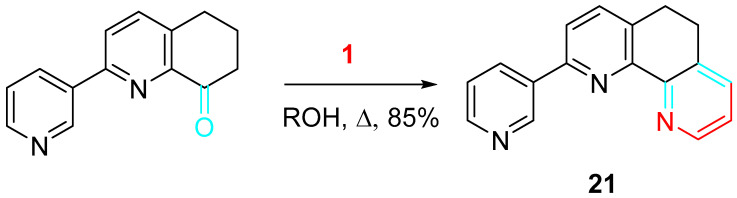
Synthesis of the dihydrophenanthroline **21**.

Moreover, substituted dihydrophenanthrolines **21** were easily obtained from 2-substituted 6,7-dihydroquinoline-8(5H)-ketones and propargylamine in alcohol at 70–130 °C. This metal-free method has the advantages of safety, cleanness and wide substrate applicability. The product can be efficiently isolated by adjusting the temperature or prolonging the reaction time (Scheme 21) [39].

**Scheme 22:**
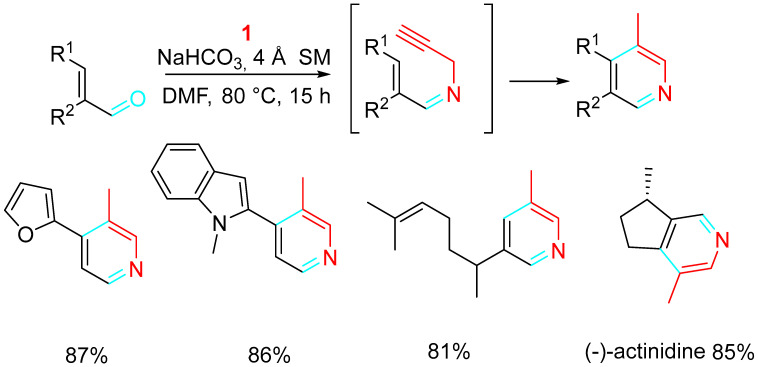
Synthesis of natural products.

The reaction of readily available α,ß-unsaturated carbonyl compounds with propargylamine provided a high atom- and pot-economy strategy for the synthesis of polyfunctionalized pyridines under metal-free conditions with relevant functional group tolerance. The exploration of bases (CsCO_3_, NaHCO_3_, NaOAc, K_2_HPO_4_, DBU) and solvents (toluene, DCE, THF, DMSO, DMF) achieved the optimization of the reaction conditions by reacting the propargylamines with the unsaturated aldehydes in DMF in the presence of NaHCO_3_ at 80 °C [40]. The application to the synthesis of a variety of natural products was reported (Scheme 22) [41].

**Scheme 23:**
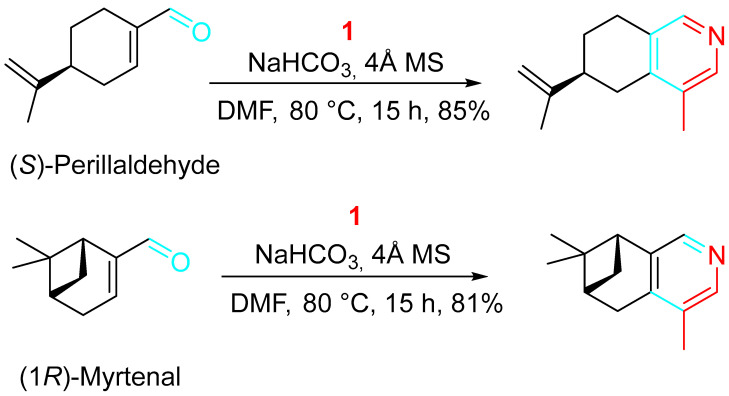
Construction of pyridines starting from (*S*)-(−)-perillaldehyde and (1*R*)-myrtenal.

The method was applied to the synthesis of pyridines from the cosmetic, flavor and fragrance agent (*S*)-(−)-perillaldehyde and the flavoring agent found in cardamom, (1*R*)-myrtenal (Scheme 23).

**Scheme 24:**
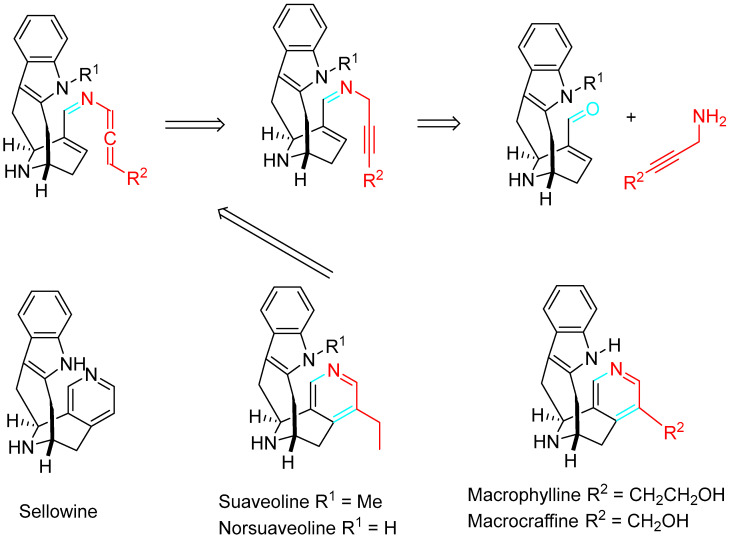
Retrosynthetic pathway of suaveoline alkaloids.

The process also achieved the total syntheses of suaveoline alkaloids (Scheme 24) [42].

**Scheme 25:**
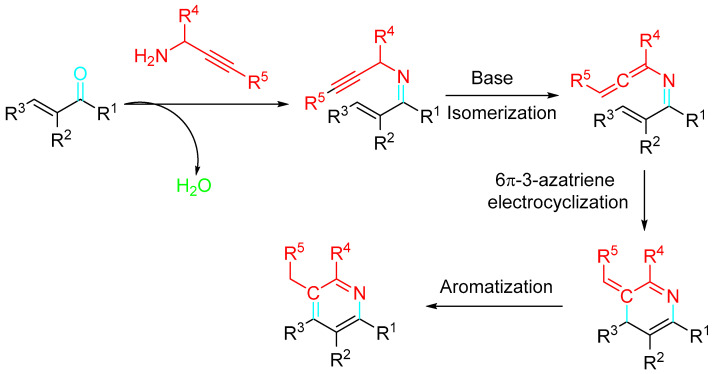
Pyridine synthesis by a 6π-3-azatriene electrocyclization.

The practicality of the protocol for the sustainable synthesis of these kinds of molecules through a tandem condensation–alkyne isomerization–6π-3-azatriene electrocyclization sequence was highlighted (Scheme 25) [43].

**Scheme 26:**
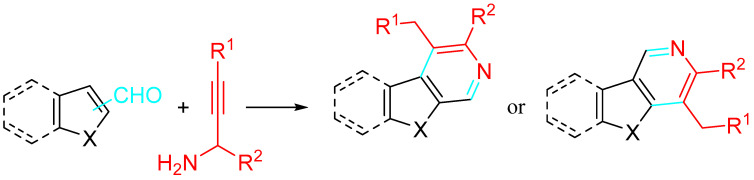
Synthesis of β- and γ-carbolines from indole aldehydes and substituted propargylic amines.

The reaction of propargylamines with (hetero)aromatic aldehydes efficiently afforded β-carbolines, γ-carbolines and other fused azaheteroaromatics under metal-free conditions (Scheme 26) [44].

**Scheme 27:**
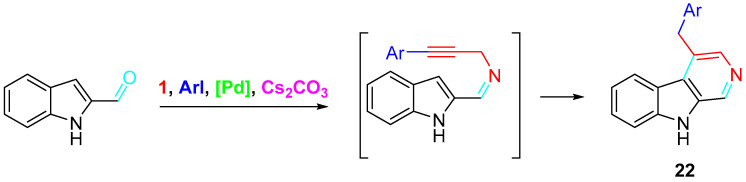
Synthesis of 4-benzylated carbolines **22**.

A one-pot, three-component method allowed the preparation of 3-substituted pyridines and carbolines **22** via copper-free, palladium-catalyzed Sonogashira cross-coupling with aryl iodides, followed by 6π-aza cyclization. This method selectively provided the fused pyridines with good yields (67–92%) (Scheme 27) [45].

**Scheme 28:**
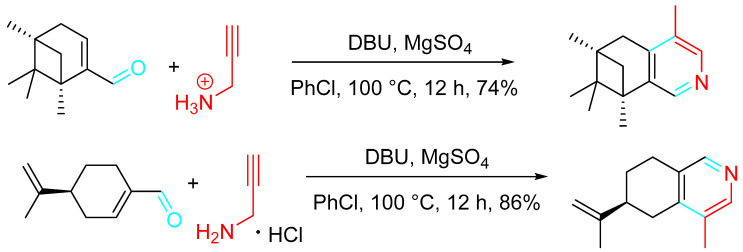
Alternative synthesis of polysubstituted pyridine derivatives from α,β-unsaturated carbonyl compounds and propargylamine hydrochloride.

Alternatively, a further preparation method for the polysubstituted pyridine derivatives comprised the employment of an α,β-unsaturated carbonyl compound and propargylamine hydrochloride as raw materials in chlorobenzene (PhCl) with the sequential addition of 1,8-diazabicyclo [5.4.0]undec-7-ene (DBU) and magnesium sulfate (MgSO_4_) (Scheme 28). The advantage of this alternative procedure is a relatively strong industrial application prospect [46].

**Scheme 29:**
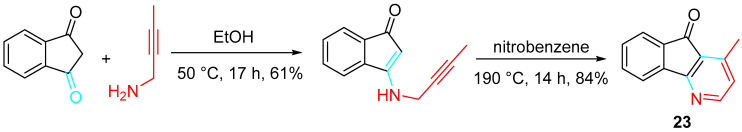
Total synthesis of onychine **23**.

Accordingly, an easy synthesis of onychine **23**, an azafluorenone alkaloid isolated from a plant of the *Annonaceae* family, was reported to occur through aza-Claisen rearrangement, tautomerization, 1,5-sigmatropic hydrogen shift, 6π-electron cyclization, and oxidation of the *N*-propargyl enamine, obtained in a yield of 61% by dehydration condensation of but-2-yn-1-amine with 1,3-indanedione (Scheme 29) [47].

**Scheme 30:**
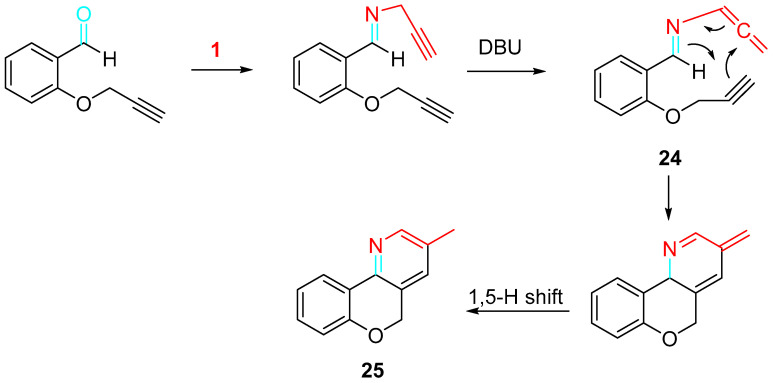
Mechanism for the formation of chromenopyridines **25**.

The sequential *O*-propargylation of aromatic hydroxyaldehydes/condensation reaction with propargylamine allowed a simple approach to the synthesis of chromenopyridine and chromenopyridinone derivatives. The intramolecular cycloaddition reaction between the alkyne and azadiene of **24**, which is formed as an intermediate, furnished the desired skeleton of chromenopyridine **25** (Scheme 30) [48].

**Scheme 31:**
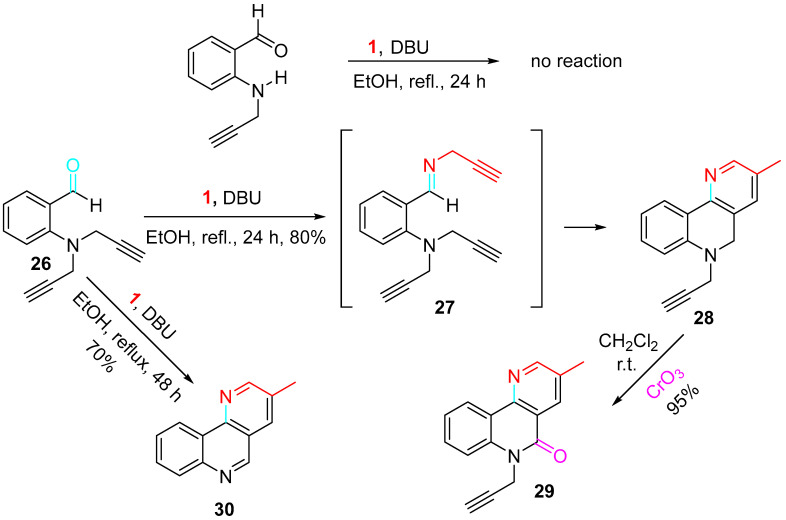
Sequential reaction of 2-(bis(prop-2-yn-1-ylamino)methyl benzaldehyde **26** with propargylamine.

Moreover, the *N*-propargylation of aromatic aminobenzaldehydes, followed by reaction with propargylamine in the presence of DBU, gave the corresponding benzo[*h*][1,6]-naphthyridines **30** (Scheme 31) [49]. The lack of reactivity of the 2-(prop-2-yn-1-ylamino)benzaldehyde was surmounted by double propargylation of the aniline derivative leading to the intermediate **27**, which cyclized in refluxing ethanol to afford the *N*-propargyl derivative **28** with 80% yield. The 3-methylbenzo[*h*][1,6]-naphthyridine **30** was isolated by increasing the reaction time to 48 h. Oxidation of **28** with CrO_3_ in pyridine in dichloromethane at room temperature gave the desired product **29** with 95% yield.

**Scheme 32:**
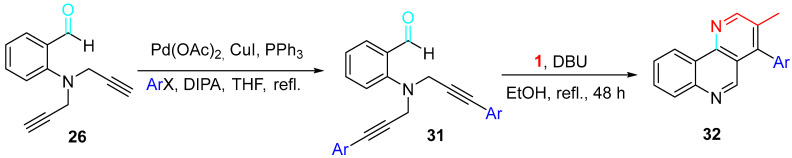
Synthesis of naphthyridines **32**.

Moreover, a variety of starting materials **31** synthesized by Sonogashira coupling reactions afforded the corresponding naphthyridine derivatives **32** by reacting with propargylamine in refluxing EtOH in the presence of DBU (Scheme 32).

**Scheme 33:**
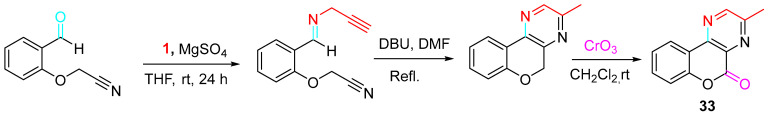
Synthesis of the chromenopyrazinone **33**.

The approach was extended to the synthesis of the chromenopyrazinone **33** (Scheme 33).

**Scheme 34:**
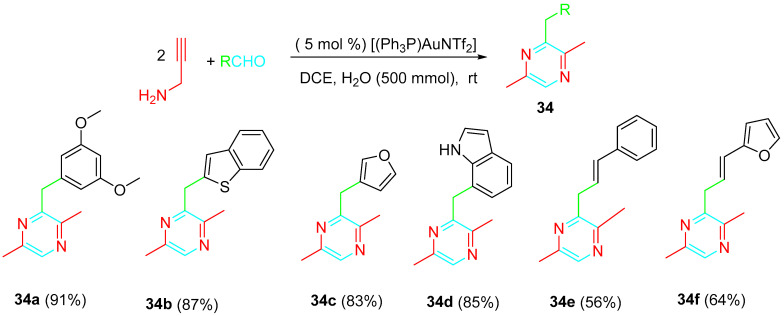
Gold-catalyzed synthesis of pyrazines **34** from the reaction of propargylamine with aldehydes.

Pyrazines **34** were also synthesized through the gold-catalyzed coupling reaction of aldehydes with propargylamine by means of a different sequential process; 1,2-dichloroethane (DCE) was the best choice as solvent. The addition of five equivalents of H_2_O under otherwise identical conditions was advantageous for the reaction outcome. The [(Ph_3_P)AuNTf_2_] catalyst (5 mol%) was identified as the most effective. The feature of the phosphine showed little effect. Any significant difference was observed by the substitution of [(Ph_3_P)AuNTf_2_] with [P(tBu)_2_(o-biphenyl)AuNTf_2_]. AuCl_3_ resulted in a less effective catalyst, while different Lewis acid catalysts, such as PtCl_2_, InCl_3_, Bi(OTf)_3_, ZnCl_2_, and AgNTf_2_, failed to afford the product. The reaction of aromatic and α,β-unsaturated aldehydes with two equivalents of propargylamine gave the corresponding pyrazine derivatives with high yields. The catalyst loading could be reduced from 5 mol% to 1 mol% without significant loss of yield of the product when the reaction was carried on a 1 gram scale (Scheme 34) [50].

**Scheme 35:**
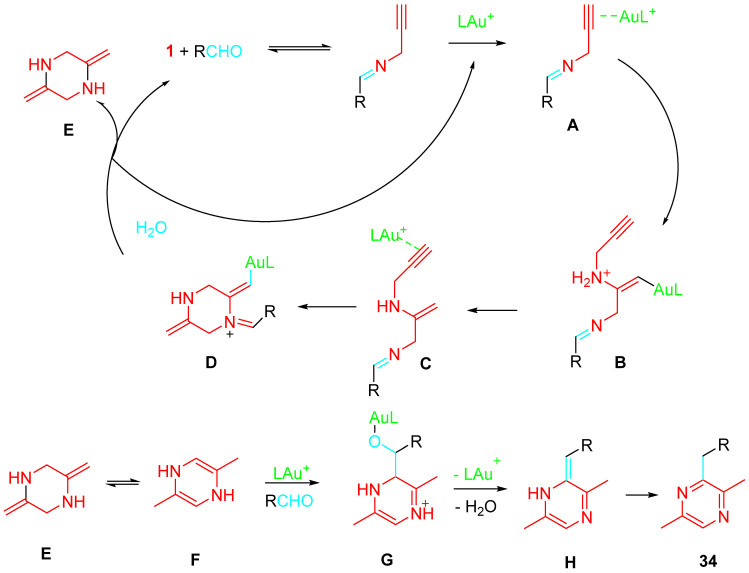
Proposed mechanism for the Au-catalyzed formation of pyrazines **34**.

The following reaction mechanism was suggested on the base of labeling experiments and density functional theory (DFT) (Scheme 35). In situ generated gold(I)-imine complex **A** undergoes a chemo- and regioselective hydroamination reaction with propargylamine to produce the intermediate **B**. The following protonolysis of the Au-C bond generates the intermediate **C**, which cyclizes to afford the intermediate **D**. This new cationic species readily releases benzaldehyde by hydrolysis, regenerating the gold catalyst and producing the 2,5-dimethylenepiperazine **E**, which readily isomerizes to its more stable 2,5-dimethyl-1,4-dihydropyrazine isomer **F**. Then, an intermolecular enamine addition from **F** towards the gold-activated benzaldehyde occurs to produce the intermediate **G**. Finally, the subsequent isomerization–aromatization sequence gives the reaction product **34**.

**Scheme 36:**
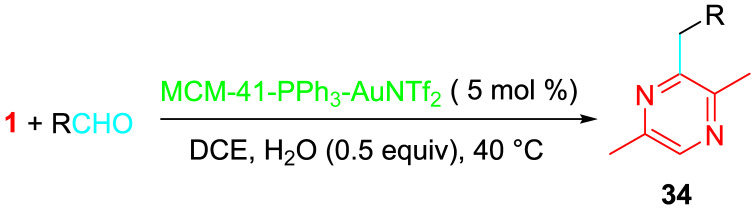
Sequential reaction of propargylamine with aldehydes catalyzed by MCM-41-immobilized phosphine gold(I) complex [MCM-41-PPh_3_-AuNTf_2_].

The heterogeneous gold(I)-catalyzed version of the cascade reaction of aldehydes with propargylamine occurred in 1,2-dichloroethane (DCE) at 40 °C under the presence of the readily available mesoporous MCM-41-immobilized phosphine gold(I) complex (MCM-41-PPh_3_-AuNTf_2_). The easy-to-prepare heterogeneous gold(I) catalyst could be recovered by filtration and recycled (Scheme 36) [51].

**Scheme 37:**
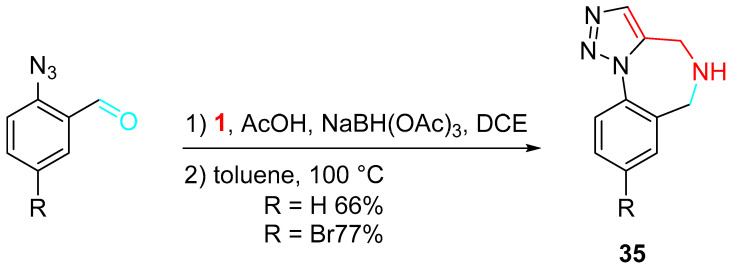
Synthesis of 1,2,3-triazolo-1,4-benzodiazepines **35**.

A variant of a sequential multicomponent assembly process (MCAPs)–cyclization approach in accord with the plan outlined in Scheme 37 was explored for preparing a variety of 1,2,3-triazolo-1,4-benzodiazepines **35** of possible medical relevance by a sequential reductive amination of 2-azidobenzaldeyde derivatives with propargylamine/intramolecular Huisgen cycloaddition [52].

**Scheme 38:**
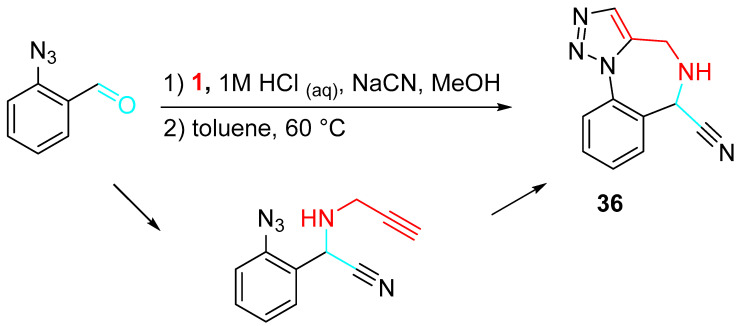
Diversely substituted 1,2,3-triazolo-1,4-benzodiazepine **36**.

A wide library was obtained through *N*-functionalizations, palladium-catalyzed cross-coupling reactions, and applications of α-aminonitrile chemistry (Scheme 38). [53]

**Scheme 39:**
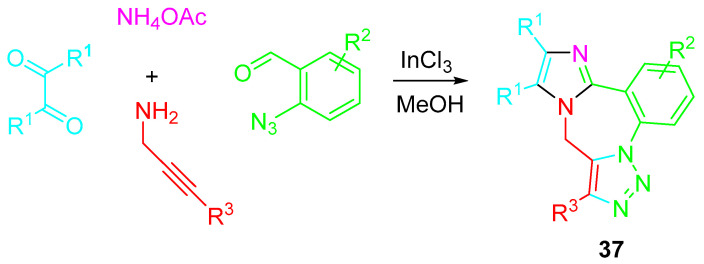
Indium-catalyzed multicomponent synthesis of 9*H*-benzo[*f*]imidazo [1,2-*d*][1,2,3]triazolo [1,5-*a*][1,4]diazepines **37**.

An atom-economical multicomponent sequential InCl_3_-catalyzed cyclocondensation/azide-alkyne 1,3-dipolar cycloaddition of 2-azidobenzaldehydes with propargylamines under the presence of α-diketone and ammonium acetate efficiently afforded the corresponding 9*H*-benzo[*f*]imidazo [1,2-*d*][1,2,3]triazolo [1,5-a][1,4]diazepines **37** (Scheme 39) [54].

## 3. Sequential Reactions of β-Aminoalkynes with Carbonyls

**Scheme 40:**
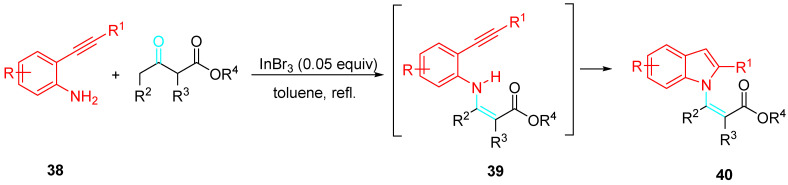
Indium-catalyzed synthesis of β-*(N*-indolyl)-α,β-unsaturated esters **40**.

Versatile β-aminoalkyne building blocks for the synthesis of nitrogen-containing heterocyclic compounds are represented by 2-alkynylanilines **38** [55,56,57]. Their sequential reaction with carbonyl derivatives was directed towards the formation of different scaffolds by changing the reaction conditions. The reaction of **38** with simple ketones or β-ketoesters selectively afforded the corresponding *N-(Z)-*alkenyl indoles **40** under the presence of InBr_3_ catalyst. The sequential reaction was considered to proceed through the activation of the β-ketoesters/formation of β-enamino esters **39**/intramolecular 5*-endo-*dig cyclization promoted by activation of the acetylene (Scheme 40) [58].

**Scheme 41:**
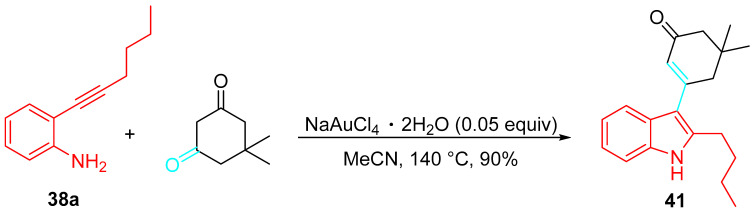
Divergent sequential gold-catalyzed cyclization/alkenylation reaction of 2-alkynylanilines with 1,3-dicarbonyl compounds.

Conversely, the divergent cyclization–alkenylation sequence to give the indole derivative **41** occurred by reacting the 2-alkynylanilines **38a** with 1,3-dicarbonyls in the presence of NaAuCl_4_·2H_2_O as the catalyst in a sealed tube (Scheme 41) [59].

**Scheme 42:**
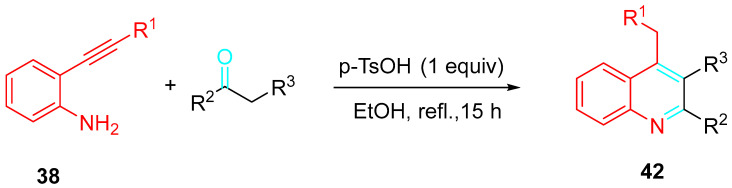
*p*-TsOH promoted synthesis of 4-alkyl-2,3-disubstituted quinolines **42**.

Moreover, reactions between readily available 2-alkynylanilines and activated ketones promoted by *p*-toluenesulfonic acid (*p*-TsOH) afforded 4-alkyl-2,3-disubstituted quinolines **42**. The features of substituents at the other end of the triple bond of 2-alkynylanilines achieved access to the 4-alkylquinolines, difficult to obtain by classical Friedländer reaction (Scheme 42) [60].

**Scheme 43:**
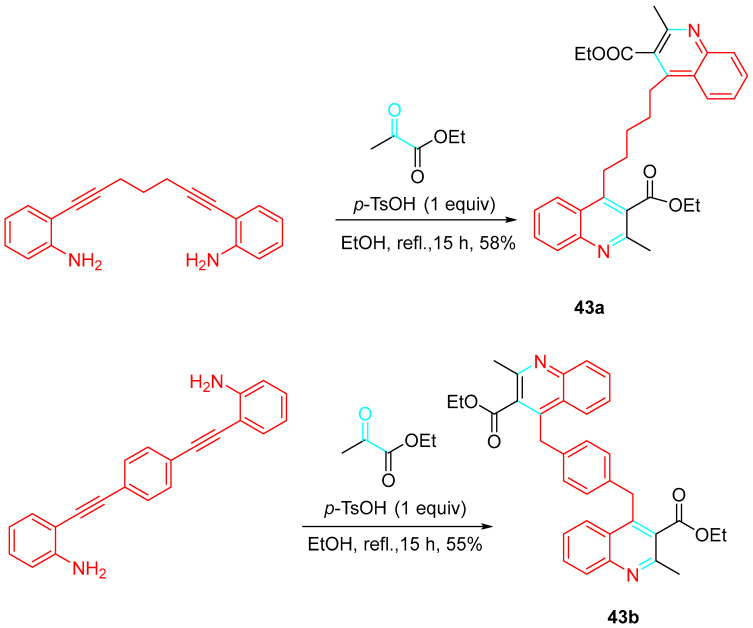
Synthesis of dimeric quinolines **43**.

The procedure also accomplished the preparation of quinoline dimers **43** with alkyl or aryl linkers at C-4 (Scheme 43).

**Scheme 44:**
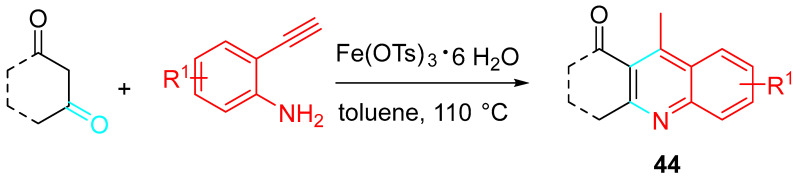
Iron(III)-catalyzed sequential condensation, cyclization and aromatization of 1,3-diketones with 2-ethynylaniline to afford 4-methyl-2,3-disubstituted quinolines **44**.

Alternatively, the one-pot synthesis of 4-methyl-2,3-disubstituted quinolines **44** was allowed by means of the inexpensive iron(III)catalyzed sequential condensation, cyclization and aromatization of 1,3-diketones with 2-ethynylaniline derivatives (Scheme 44) [61].

**Scheme 45:**
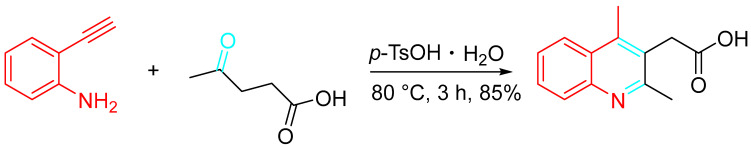
Sequential reaction of levulinic acid with 2-ethynylaniline under solventless conditions.

Furthermore, a cost-effective *p*-TsOH promoted synthetic strategy for the synthesis of substituted quinolines was explored by the reaction between levulinic acid with different 2-alkynylanilines under mild metal-free solventless conditions (Scheme 45) [62].

**Scheme 46:**
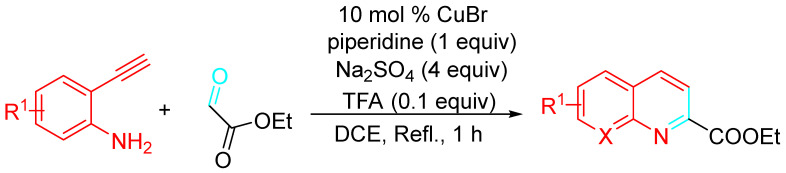
Copper(I)-catalyzed synthesis of 2-acylquinolines.

The combination of CuBr and trifluoroacetic acid (TFA) directly afforded the corresponding quinolines by reacting the 2-ethynylaniline with ethyl glyoxylate (Scheme 46) [63].

**Scheme 47:**
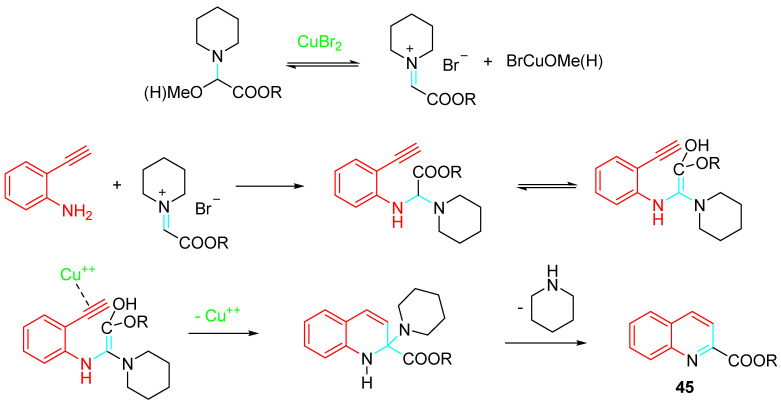
Copper-catalyzed synthesis of quinolines **45** from ethynylaniline and *N*, *O*-acetals.

*N*,*O*-acetals also functioned as a C1 part leading to the preparation of quinoline derivatives **45** according to the following path (Scheme 47) [64].

**Scheme 48:**
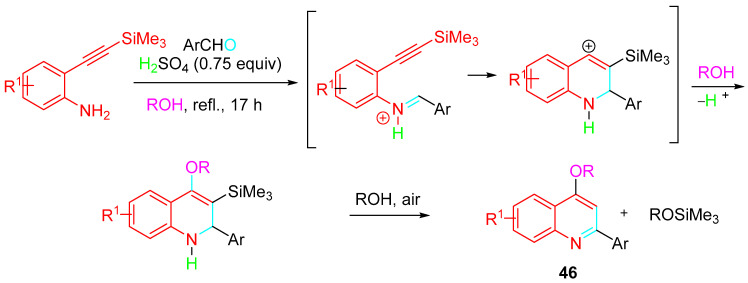
Synthesis of 4-alkoxy-2-arylquinolines **46**.

A three-component, one-pot sulfuric acid-mediated reaction of 2-(2-(trimethylsilyl) ethynyl)anilines with arylaldehydes in alcohol efficiently provided 4-alkoxy-2-arylquinolines **46** (Scheme 48) [65].

**Scheme 49:**
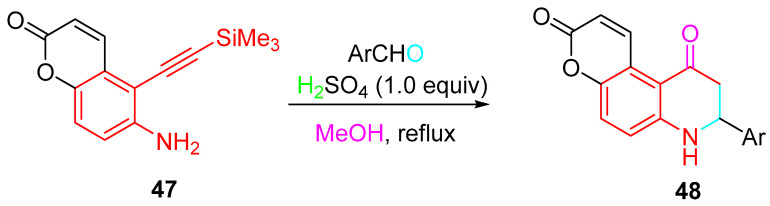
Condensative cyclization of 6-amino-5-[(trimethylsilyl)ethynyl]-2*H*-chromen-2-one **47**.

This strategy was extended to afford the unusual formation of 8-aryl-8,9-dihydro-3*H*-pyrano [3,2-*f*]quinoline-3,10(7*H*)-dione derivatives **48** with good yields by condensative cyclization of 6-amino-5-[(trimethylsilyl)ethynyl]-2*H*-chromen-2-one **47** with aromatic aldehydes (Scheme 49) [66].

**Scheme 50:**
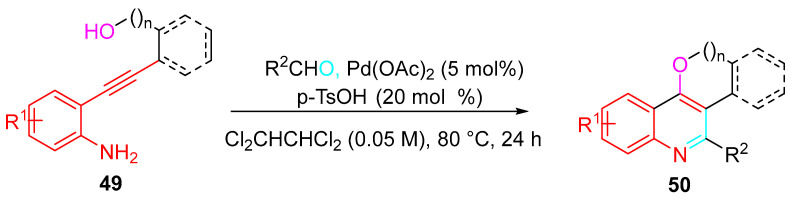
Synergistic effect of Pd(II) and acid catalysts on the synthesis of ring-fused quinolines.

It was envisioned that the in situ generated *N*-(2-alkynylphenyl)imine might be cyclized to give ring-fused quinoline derivatives. Indeed, a tandem reaction of 2-alkynylanilines **49** with aldehydes catalyzed by the combination of Pd(OAc)_2_ and *p*-TsOH allowed the regioselective synthesis of ring-fused quinolines **50** (Scheme 50) [67].

**Scheme 51:**
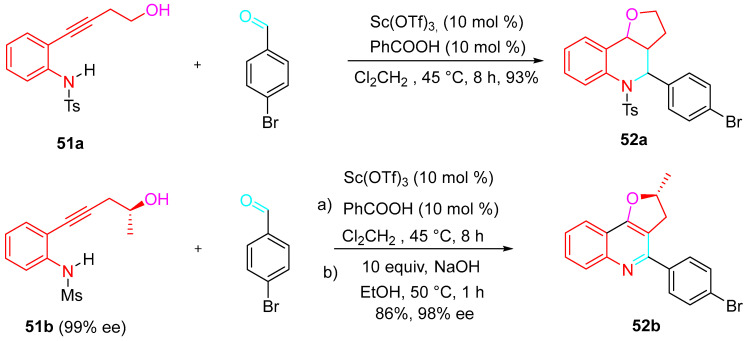
Sc(OTf)_3_-catalyzed bicyclization of 2-alkynylanilines with aldehydes.

Moreover, a Sc(OTf)_3_-catalyzed tandem aza-Prins cyclization reaction of 2-alkynylaniline derivatives **51** with aldehydes afforded under mild reaction conditions fused tricyclic derivatives **52**. Interestingly, when the enantiopure optically active 2-alkynylaniline (*R*)-**51b**, having a central chirality (99% ee), was subjected to the optimized reaction conditions followed by subsequent treatment with NaOH, the quinoline derivative (*R*)-**52b** was obtained directly (86% yield, 98% ee) (Scheme 51). Six- and seven-membered oxacyclo-fused products were also easily synthesized [68].

**Scheme 52:**
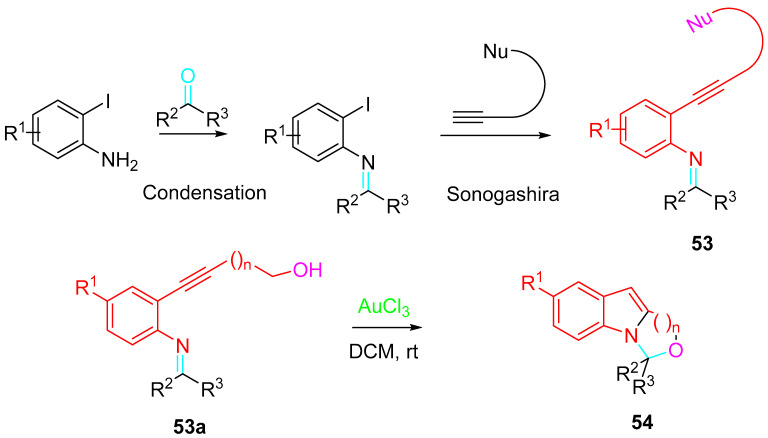
Gold-catalyzed synthesis of polycyclic frameworks **54**.

It is worth noting that the *N*-(2*-*alkynylphenyl) imines **53** readily available by means of condensation of 2*-*iodoanilines with ketones or aldehydes followed by Sonogashira coupling with acetylenes were prone to undergo different sequential processes. Ring-fused indoles **54** were obtained from *N*-(2*-*alkynylphenyl) imines **53a** with high yields under mild conditions and in the presence of a gold (III) as a catalyst (Scheme 52) [69].

**Scheme 53:**
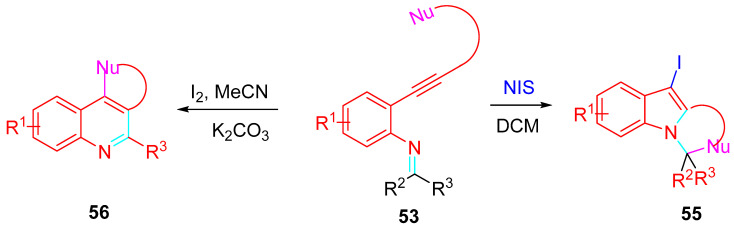
Iodonium-induced tandem cyclization of *N*-(2*-*alkynylphenyl) imines **53**.

Furthermore, the *N*-(2*-*alkynylphenyl) imines intermediates **53** treated with *N*-iodosuccinimide (NIS) in DCM induced novel iodonium mediated domino reaction cascades, which provided ring-fused indole compounds **55** or simply by changing the reaction conditions ring-fused quinoline compounds **56** (Scheme 53) [70].

**Scheme 54:**
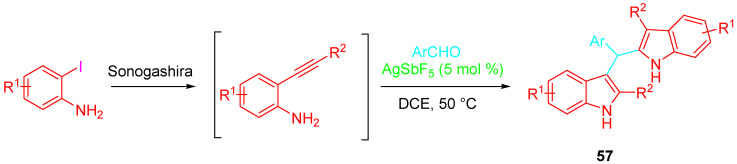
One-pot synthesis of 2,2′-disubstituted diindolylmethanes **57**.

The application of the methodology to the synthesis of iodoquinolones from suitable *N*-(2-alkynylphenyl)imine [71] as well as to the construction of polycyclic indole derivatives through the [3 + 2] cycloaddition of metal-containing azomethine ylides generated from *N*-(*o*-alkynylphenyl)imine derivatives and W(CO)_5_(L) was also reported [72]. A different sequential cycloisomerization/C3-functionalization of the in situ generated 2-alkynylanilines via Sonogashira coupling of 2-iodoanilines determined a one-pot synthesis of 2,2′-disubstituted diindolylmethanes **57** (Scheme 54) [73].

**Scheme 55:**
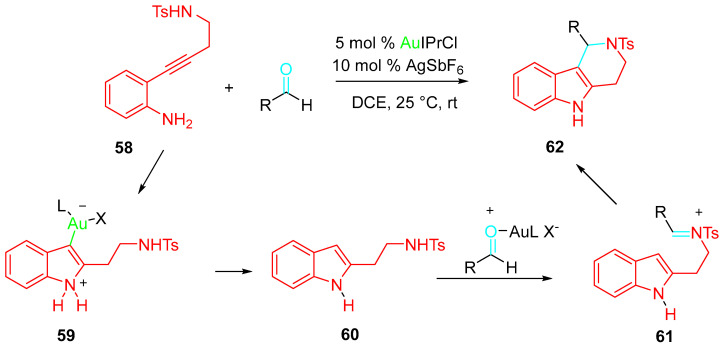
Gold-catalyzed synthesis of 1-substituted 2-tosyl-2,3,4,5-tetrahydropyrido [4,3-*b*]indoles **62**.

A one-pot strategy for the synthesis of 1-substituted 2-tosyl-2,3,4,5-tetrahydropyrido [4,3-*b*]indole scaffolds **62** through a sequential gold-catalyzed hydroamination/Pictet–Spengler cyclization of 2-(4-aminobut-1-yn-1-yl)aniline with aldehydes was demonstrated (Scheme 55) [74]. The initial π-coordination of cationic Au(I) species with the alkyne moiety of 2-(4-aminobut-1-yn-1-yl)aniline **58** forms a π-complex that gives the cyclic intermediate **59**. The following protodemetalation affords the isotryptamine **60**. Subsequently, activation of aldehyde by Au(I) species followed by an intramolecular nucleophilic addition of indole moiety of **60** to a highly reactive *N-*sulfonyliminium intermediate **61** provides the tetrahydropyridoindole **62** with regeneration of the catalyst. Ag(I) also promotes the Pictet–Spengler reaction.

**Scheme 56:**
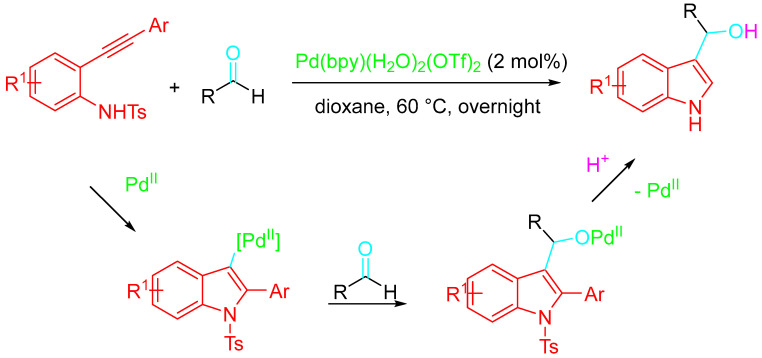
Palladium-catalyzed synthesis of *N*-tosyl-3-hyroxymethyl indoles.

The sequential aminopalladation of *N*-tosyl-2-arylethynylanilines followed by the addition to the carbonyl group of an aldehyde as the quenching step of the carbon–palladium bond gave corresponding 3-hyroxymethyl indole derivatives with good yields. Cationic palladium complexes bearing bipyridine or dppp as ligands resulted in suitable catalysts, and the best conditions were observed by carrying out the reaction in dioxane at 60 °C in the presence of the catalyst Pd(bpy)(H_2_O)_2_(OTf)_2_ (Scheme 56) [75].

**Scheme 57:**
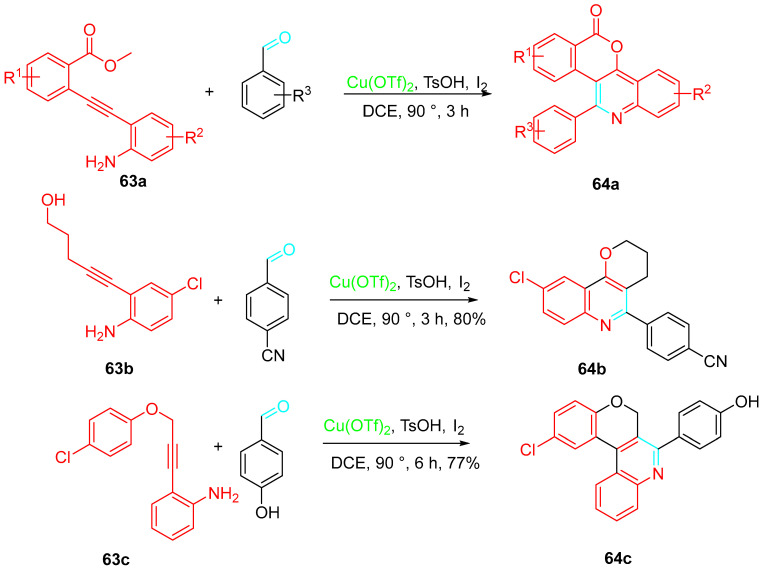
Cu(OTf)_2_-catalyzed synthesis of quinoline-annulated polyheterocyclic frameworks **64**.

A Cu(OTf)_2_-catalyzed intramolecular radical cascade reaction efficiently enabled the synthesis of quinoline-annulated compounds **64** [76]. The method represents an effective route to natural products and a variety of drug-like libraries (Scheme 57).

**Scheme 58:**
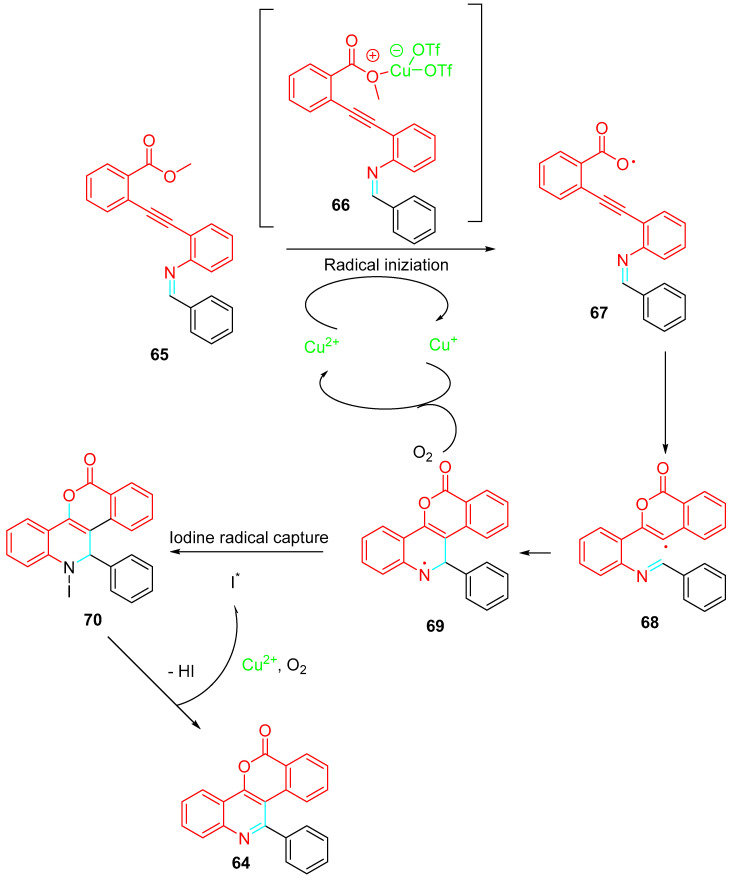
Plausible reaction mechanism.

The proposed mechanism for the synthesis of the polyheterocyclic scaffolds is shown in the following Scheme 58. The intermediate **65** undergoes a copper salt-promoted one-electron oxidation to generate the intermediate **66**. Subsequent radical addition into the C-C bond of **67** affords the radical **68**, which cyclizes to give the radical **69**. Finally, trapping of the nitrogen radical by the iodine radical generated from the oxidation of iodide affords the complex **70**, which after iodide elimination furnishes the product **64**.

**Scheme 59:**
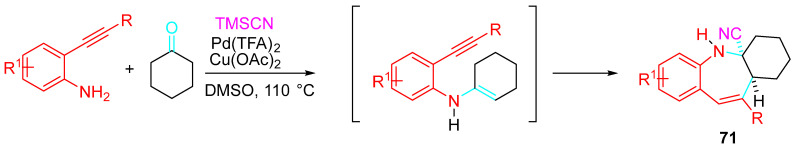
Palladium-catalyzed synthesis of 1-benzoazepine carbonitriles **71**.

A regio- and stereoselective three-component, one-pot cascade reaction involving an imination–annulation–cyanation sequence was achieved by combining palladium(II) trifluoroacetate and copper(II) acetate with the readily available 2-alkynylanilines, cyclic ketones and trimethylsilyl cyanide in dimethyl sulfoxide to efficiently afford the corresponding 1-benzoazepine carbonitrile derivatives **71** (Scheme 59) [77].

**Scheme 60:**
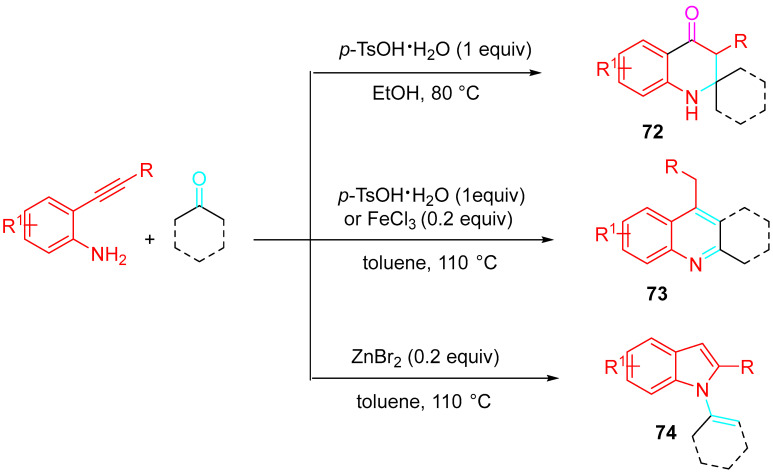
Product-selectivity control of the sequential reaction of 2-alkynylaniline with ketones.

The construction of spirocyclic quinolones **72**, which are difficult to synthesize through traditional methodologies, was explored by selectively directing the reaction of 2-alkynylanilines with ketones under suitable reaction conditions. Interestingly, the same starting reagents selectively produced the quinolines **73** or the *N-*alkenyl indoles **74** under different reaction conditions (Scheme 60) [78].

**Scheme 61:**
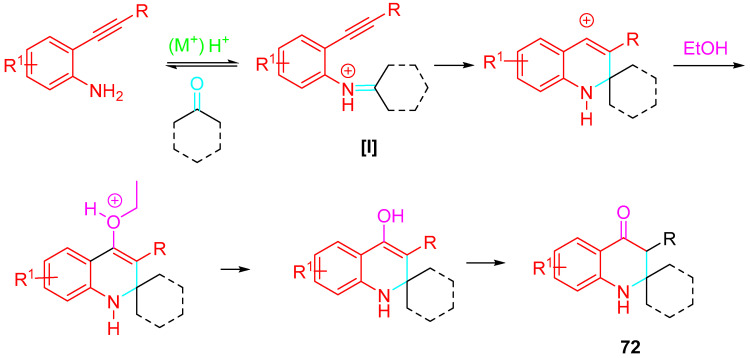
Proposed mechanism for the synthesis of quinolinones **72**.

Very likely, the condensation reaction under the Brønsted acid-mediated conditions in EtOH led to the iminium ion intermediate **[I]** which undergoes *aza*-Prins to afford an intermediate, which—after quenching by ethanol, hydrolysis and tautomerization reactions—generated the quinolinone **72** (Scheme 61).

**Scheme 62:**
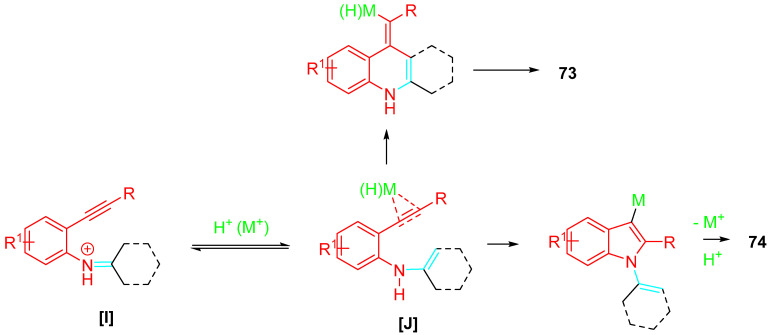
Proposed mechanism for the synthesis of quinolines **73** and *N*-alkenylindoles **74**.

Conversely, isomerization of the iminium ion intermediate **[I]** to the intermediate **[J]** should lead selectively to the indoles **74** via a 5*-endo-*dig cyclization or to the quinoline **73** via a regiodivergent 6*-exo-*dig cyclization (Scheme 62).

**Scheme 63:**
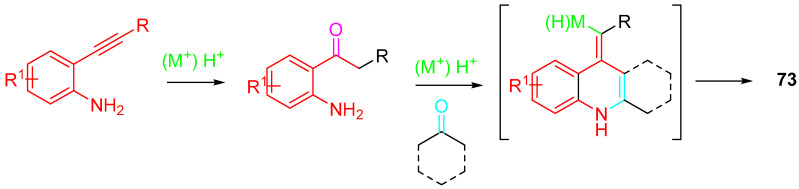
Alternative mechanism for the synthesis of quinolines **73**.

Alternatively, the quinolines **73** could be generated from the 2-aminoaryl ketone obtained by the fast hydration reaction of the 2-alkynylaniline, both in the presence of a stoichiometric amount of *p*-TsOH·H_2_O or 0.2 equiv. of FeCl_3_ in toluene at 110 °C (Scheme 63).

**Scheme 64:**
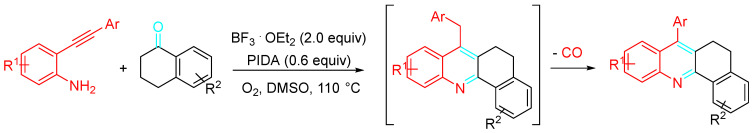
Synthesis of acridine derivatives.

Interestingly, the features of the substituent bonded at the terminal position of the triple bond of the 2-alkynylaniline and of the reaction medium determined the reaction path. Internal alkynes allowed the *p*-TsOH·H_2_O-mediated preparation of quinolones **72** in EtOH at reflux or the formation of the quinolines **73** in toluene at 110 °C both in the presence of a stoichiometric amount of *p*-TsOH·H_2_O or FeCl_3_ as the catalyst. Conversely, the ZnBr_2_-catalyzed reaction in toluene at 110 °C of the same internal alkyne derivatives gave only the *N*-alkenylindoles **75**. The presence of a trimethylsilyl group or the absence of substituents at the terminal position of the starting aminoalkyne resulted in the formation of the corresponding quinolines. The Lewis acid-promoted reaction of 2-arylethynylanilines with α-tetralones under the presence of the strong oxidant (diacetoxyiodo)benzene (PIDA) triggered a decarbonylative cascade approach to the synthesis of acridines (Scheme 64) [79].

**Scheme 65:**
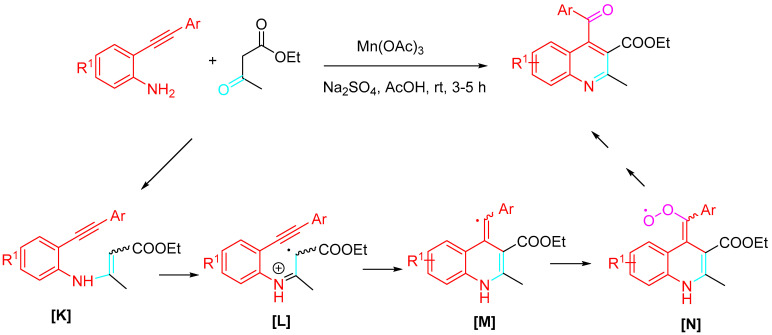
Mn(OAc)_3_-mediated synthesis of 2-substituted quinolines from 2-alkynylanilines and β-ketoesters.

A general procedure was described for the direct preparation of 2-methyl-3,4-diacylquinolines in short periods under mild reaction conditions by means of the Mn(OAc)_3_ mediated reaction in acetic acid of 2-alkynylanilines with β-ketoesters (Scheme 65). The presence of molecular oxygen and Na_2_SO_4_ as desiccant displayed a key role. It was suggested that the formation of the radical intermediate **[L]** generated by Mn(OAc)_3_ oxidation of the enaminoate **[K]** underwent a fast 6-*exo*-dig cyclization, resulting in the formation of the vinyl intermediate **[M]**. The subsequent addition of molecular oxygen to the vinyl radical should provide a peroxy radical species **[N]**, which—after protonation and loss of water—produced the 2-methyl-3,4-diacylquinolines [80].

**Scheme 66:**
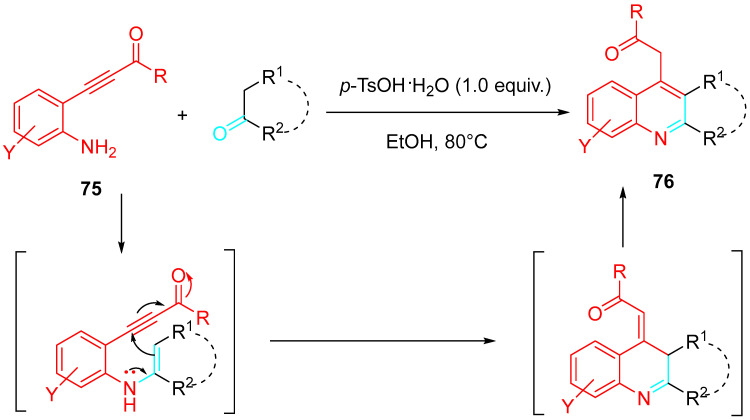
Sequential amination–annulation–aromatization reactions of β-(2-aminophenyl)-α,β-ynones **75** with enolizable ketones.

Moreover, the sequential Brønsted acid mediated reaction with enolizable ketones of the starting aminoalkynes β-(2-aminophenyl)-α,β-ynones **75** in EtOH resulted in an efficient approach to only polycyclic quinolines **76** (Scheme 66) [81].

**Scheme 67:**
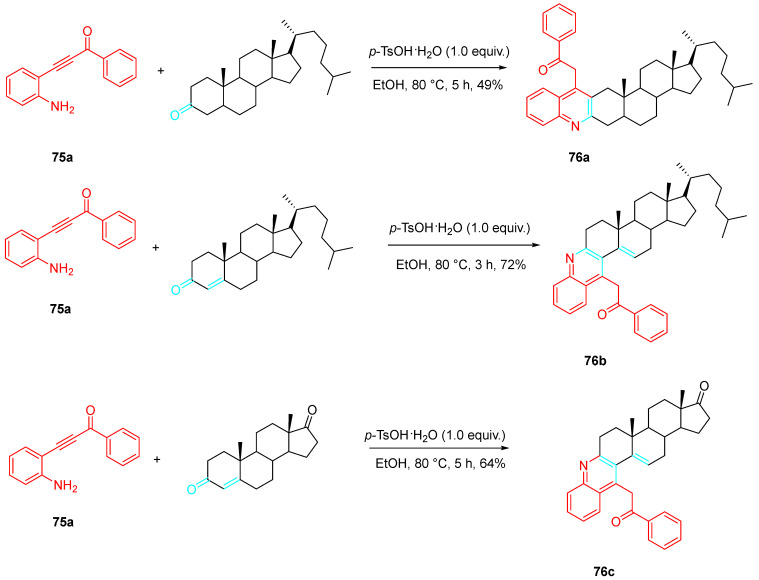
Product-selectivity control in the synthesis of polycyclic steroidal quinolines **76**.

Steroids bearing a simple ketone group at position 3, such as 5α-cholestan-3-one, formed only the corresponding linear cholestanoquinoline derivative in moderate yield. On the contrary, the optimized methodology allowed the divergent generation of the angular quinoline derivative, whose synthesis is generally considered more challenging and demanding, from 3-keto-Δ^4^-polycyclic steroidal derivatives. Interestingly, with steroidal dicarbonyl derivatives, the condensation reaction took place selectively only on the conjugated carbonyl group at position 3, leaving the ketone group at position 17 unreacted (Scheme 67). A- and D-ring fused steroidal quinoline analogues represent potential as antibacterial agents [82].

**Scheme 68:**
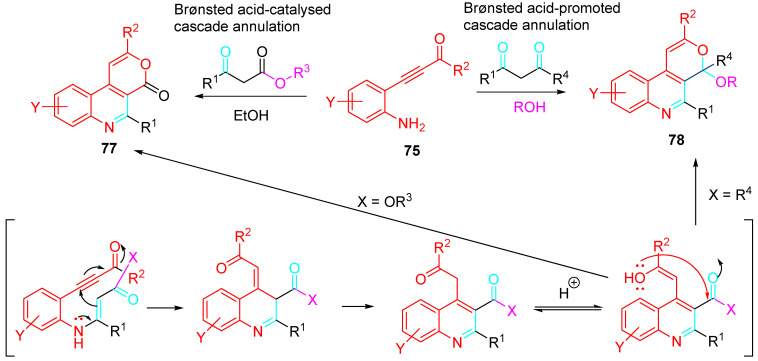
Domino reactions of β-(2-aminophenyl)-α,β-ynones with 1,3-dicarbonyls.

The Brønsted acid-promoted reaction of β-(2-aminophenyl)-α,β-ynones with ketones was expanded to activated carbonyl compounds, such as β-ketoesters and β-diketones. The carbonyl group at position 3 of the quinoline nucleus could further react with the other keto functionality in the alkyl substituent at position 4, generating an additional [3,4]-fused six-membered ring whose structure depends on the type of β-dicarbonyl compound used. Indeed, for β-ketoesters, a thorough screening of reaction conditions revealed that catalytic amounts of *p*-TsOH·H_2_O were sufficient to efficiently promote a cascade double cyclization leading to 4*H*-pyrano [3,4-*c*]quinoline-4-one derivatives **77**. On the contrary, with β-diketones, a stoichiometric amount of *p*-TsOH·H_2_O triggered a three-component reaction, involving a molecule of the alcoholic solvent to afford **78**. Both procedures appear to be simple and versatile, and are expected to be of great impact because of the multiple potential applications of the obtained organic compounds (Scheme 68) [83].

**Scheme 69:**
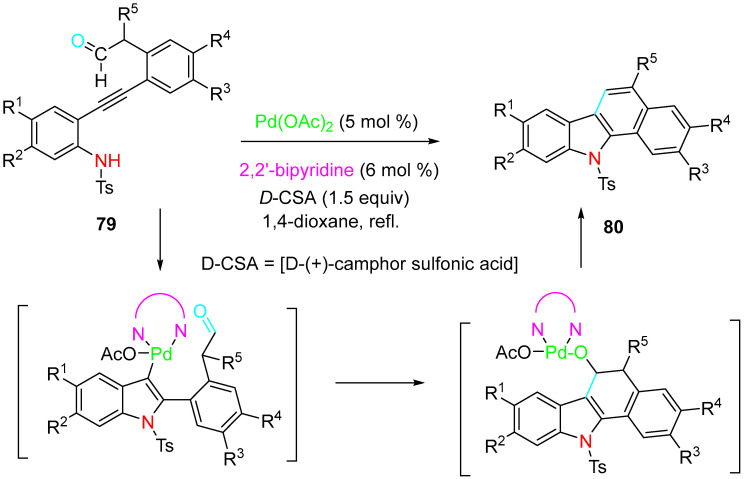
Sequential aminopalladation of β-amino alkynes **79**.

A sequential aminopalladation of β-amino alkyne derivatives **79**, followed by intramolecular nucleophilic addition of the generated carbon–palladium bond to a tethered aldehyde group, accomplished the synthesis of a variety of benzo[*a*]carbazoles **80** with remarkable diversification (Scheme 69) [84].

**Scheme 70:**
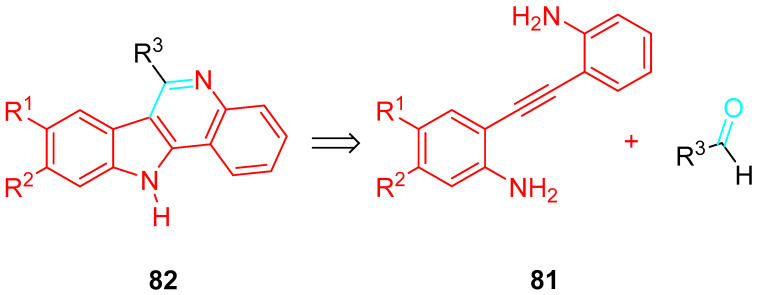
Retrosynthetic assembly of *11H*-indolo [3,2-*c*]quinolines.

The ongoing research activity devoted to the synthesis of indole derivatives encouraged the exploration of a highly flexible approach to *11H*-indolo [3,2-*c*]quinolines **82** according to the retrosynthetic Scheme 70.

**Scheme 71:**
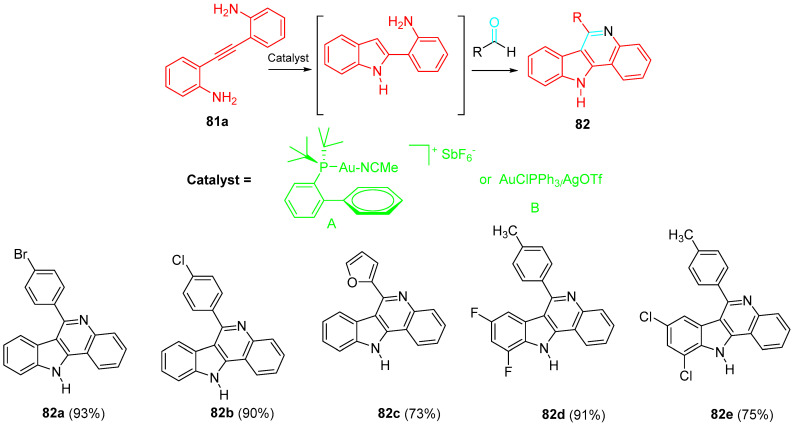
Gold-catalyzed synthesis of *11H*-indolo [3,2-*c*]quinoline **82**.

Subsequently, the selective build-up of the *11H*-indolo [3,2-*c*]quinoline **82** was carried out through a two-step, one-pot, gold-catalyzed reaction in CH_3_CN at room temperature of aldehydes with the 2,2′-(ethyne-1,2-diyl)dianiline derivative **81a** as the starting β-aminoalkynes, which was cyclized under the presence of Au catalyst (5 mol%). Then, the aldehyde (2 equiv.) was added and the reaction mixture was stirred till completion. This alternative highly regioselective protocol is of wide applicability in mild neutral reaction conditions (Scheme 71) [85].

**Scheme 72:**
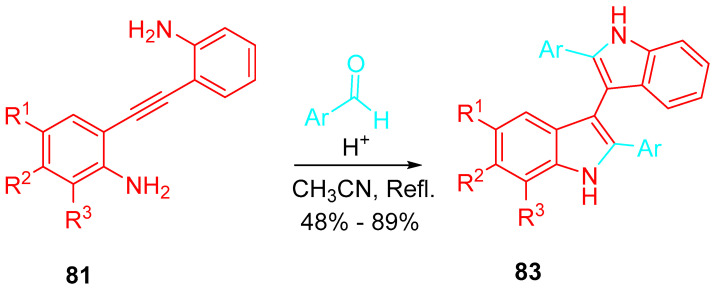
Brønsted acid-catalyzed synthesis of 2,2′-disubstituted *1H*,*1′H*-3,3′-biindoles **83**.

Surprisingly, the reaction of inexpensive aryl(heteroaryl)aldehydes with the same starting 2,2′-(ethyne-1,2-diyl)dianiline derivatives **81** in the presence of a catalytic amount of HCl achieved the synthesis of 2,2′-disubstituted-*1H*,*1*′*H*-3,3′-biindoles **83** (Scheme 72) [86].

**Scheme 73:**
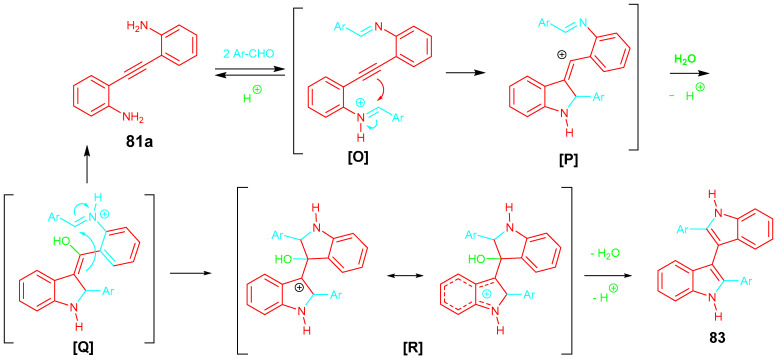
Proposed mechanism.

Accordingly, a class of double D-π-A branched organic dyes based on 2,2′-disubstituted-*1H*,*10H*-3,3′-biindole moiety has been synthesized as photosensitizers for dye-sensitized solar cells [87]. Alternatively, the 2,2′-disubstituted 3,3′-biindoles were obtained by using an acidic deep eutectic solvent (DES) able to exploit double activity, i.e., solvent and Brønsted acid (BA) catalyst under microwave heating at 70 °C [88]. Very likely, the BA promotes the formation of the iminium ion **[O]** by reaction of **83** with two equiv. of the aldehyde. The iminium ion **[O]** undergoes an aza-Prins type 5-*exo-*dig cyclization to the intermediate **[P]** quenched by the nucleophilic addition of water to give intermediate **[Q]**. The subsequent cyclization generates the stabilized benzylic carbocation intermediate **[R]** which provides the desired biindoles **83** by the loss of a molecule of water and proton regeneration (Scheme 73).

**Scheme 74:**
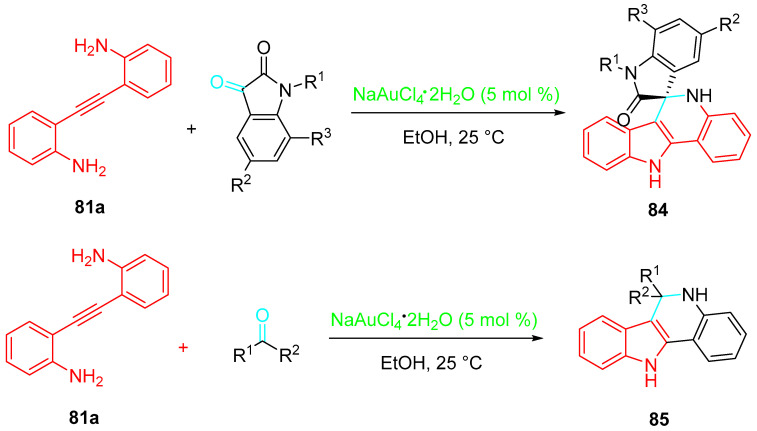
Gold-catalyzed reaction of 2-[(2-aminophenyl)ethynyl]phenylamines **81** with isatins and ketones.

Conversely, the tandem gold-catalyzed 5-*endo*-dig/spirocyclization of 2-[(2-aminophenyl)ethynyl]phenylamines **81** with isatins regioselectively afforded the corresponding 5′,11′-dihydrospiro-[indoline-3,6′-indolo [3,2-*c*]quinolin]-2-one derivatives **84** with good yields at room temperature. The reaction with ketones gave the 6,6-disubstituted-6,11-dihydro-5*H*-indolo [3,2-*c*]-quinolones **85** (Scheme 74) [89].

**Scheme 75:**
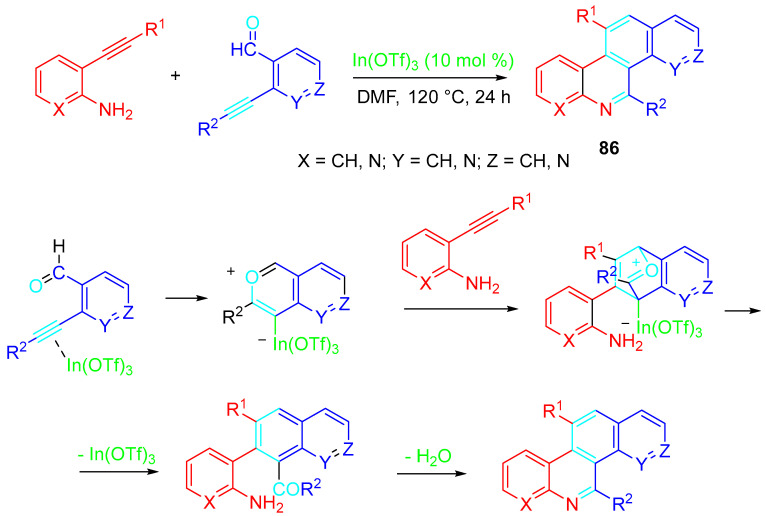
Indium(III)-catalyzed sequential reaction of 2-alkynylanilines with 2-alkynylbenzaldehydes.

A sequential intramolecular nucleophilic attack–intermolecular cycloaddition–dehydration reaction addressed the synthesis of ring-condensed heteroaromatic compounds **86** starting from 2-alkynylbenzaldehydes and 2-alkynylanilines in the presence In(OTf)_3_ catalyst (Scheme 75) [90].

**Scheme 76:**
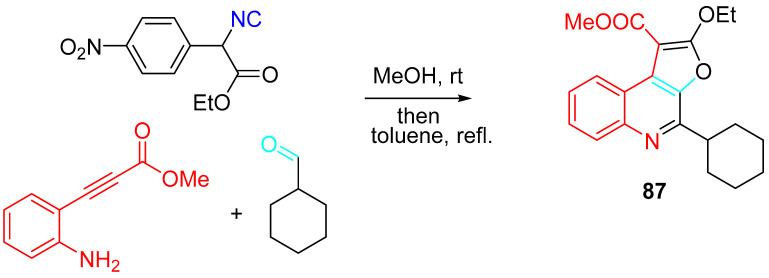
Synthesis of 2-alkoxyfuro [2,3-*c*]quinolones **87**.

An intriguing three-component reaction of 2-alkynylanilines, aldehydes and α-(4-nitrophenyl)-α-isocyanoacetates in methanol at room temperature, followed by addition of toluene and heating to reflux, provided the polysubstituted furo[2,3-*c*]quinolines **87** with satisfactory yields (Scheme 76) [91].

**Scheme 77:**
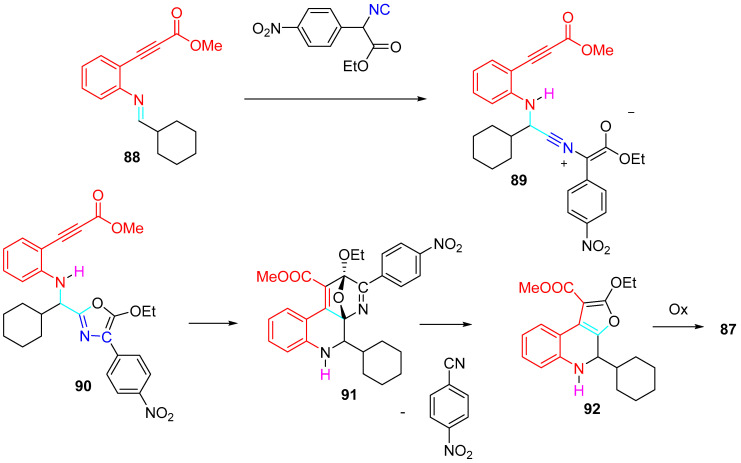
Proposed mechanism.

The reaction of the in situ formed imine intermediate **88** with α-isocyanoacetates produces the 5-alkoxyoxazoles **89**, which—through an intramolecular Diels–Alder cycloaddition between the oxazole and the tethered triple bond—generate oxa-bridged heterocycles **90**. Their subsequent fragmentation by a retro Diels–Alder process furnishes derivatives **91** and benzonitrile. Finally, oxidation mediated by atmospheric oxygen leads to the target aromatic 2-alkoxyfuro [2,3-*c*]-quinolones **87** (Scheme 77).

**Scheme 78:**
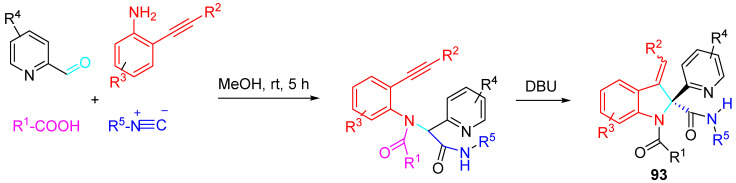
Sequential multicomponent approach to 2,2-disubstituted 3-methyleneindolines **93**.

Similarly, the three-component reaction of an aminopentanoate, an aldehyde, and an α-isocianoacetamide produced in a one-pot process the 4,5,6,7-tetrahydrofuro [2,3-*c*]pyridines by simply heating the solution in toluene in the presence of ammonium chloride. [92] A base-promoted post-Ugi 5-exo-dig “Conia-ene”-type cyclization efficiently afforded a variety of 2,2-disubstituted 3-methyleneindoline derivatives **93** (Scheme 78) [93].

## 4. Sequential Reactions of γ- and δ-Aminoalkynes with Carbonyls

**Scheme 79:**
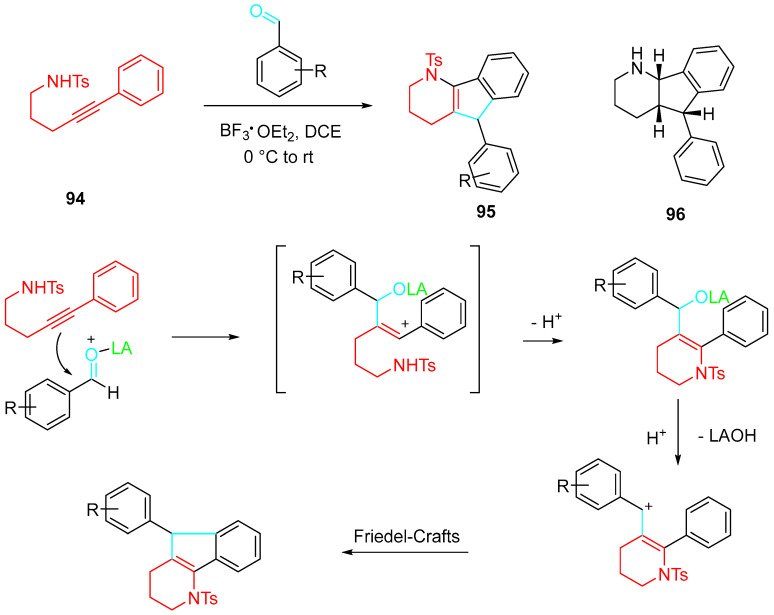
Synthesis of 1-tosyl-2,3,4,5-tetrahydro-*1H*-indeno [1,2-*b*]-pyridines **95**.

Sequential reactions of of γ- and δ-aminoalkynes with carbonyls have been less investigated. A library of 1-tosyl-2,3,4,5-tetrahydro-1H-indeno [1,2-*b*]-pyridines **95** has been established by cascade cyclization/Friedel–Crafts reaction of 4-methyl-*N*-(pent-4-yn-1-yl)benzenesulfonamides **94** and aldehydes with good yields. The reaction was performed by using 2 equiv. of BF_3_·OEt_2_ in 1,2-dichloroethane (DCE). Worse results were obtained under the presence of different Lewis and Brønsted acids or metal triflates. Both electron-withdrawing and electron-donating groups in the aromatic ring of the aldehyde were tolerated. The methodology was applied to the total synthesis of the antidepressant agent (±)-5-phenyl-2,3,4,4*a*,5,9*b*-hexahydro-*1H*-indeno [1,2-*b*]pyridine **96** (Scheme 79) [94].

**Scheme 80:**
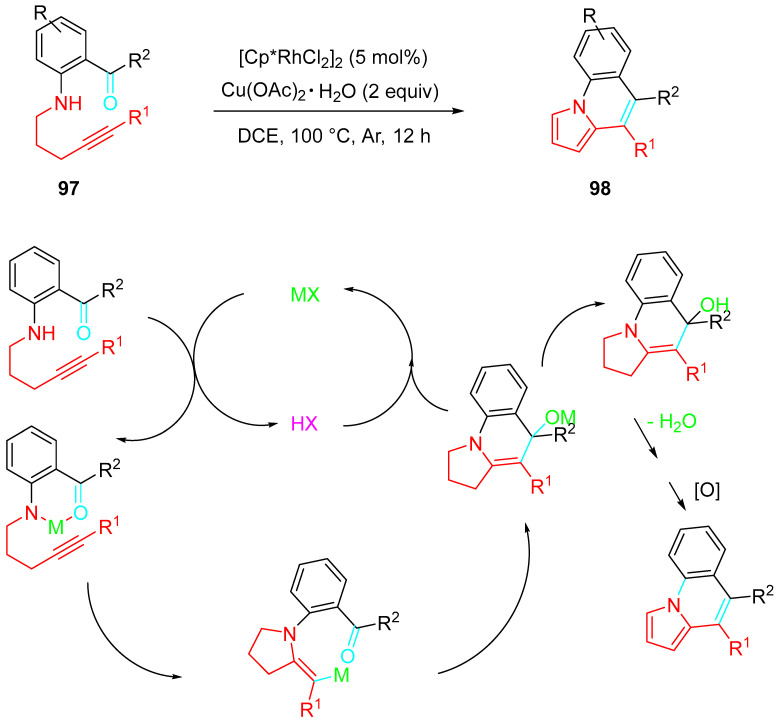
Synthesis of pyrrolo[1,2-*a*]quinolines **98**.

The sequential rhodium(III)-catalyzed intramolecular annulation/aromatization of *o-*alkynyl amino aromatic ketones **97** achieved a one-pot building up of the pyrrolo[1,2-*a*]quinolines **98**. [Cp*RhCl2]2 (Cp* = *η*^5^-1,2,3,4,5-pentamethylcyclopentadienyl) resulted in a more effective catalyst under the presence of Cu(OAc)_2_·H_2_O as the oxidant. The reaction did not occur under an air atmosphere. DCE was the solvent of choice, and inferior yields of the product were isolated when the reaction was conducted in 1,4-dioxane, acetonitrile, p-xylene, methanol, or acetic acid. The strategy provides a complementary synthetic method for the construction of 4-aryl-5-alkylpyrrolo[1,2-*a*]quinolines or those containing different aryl substituents at 4,5-positions, which are difficult to prepare by the conventional methods. The protocol could be scaled up and allowed the synthesis of challenging products suitable for further elaboration (Scheme 80) [95].

**Scheme 81:**
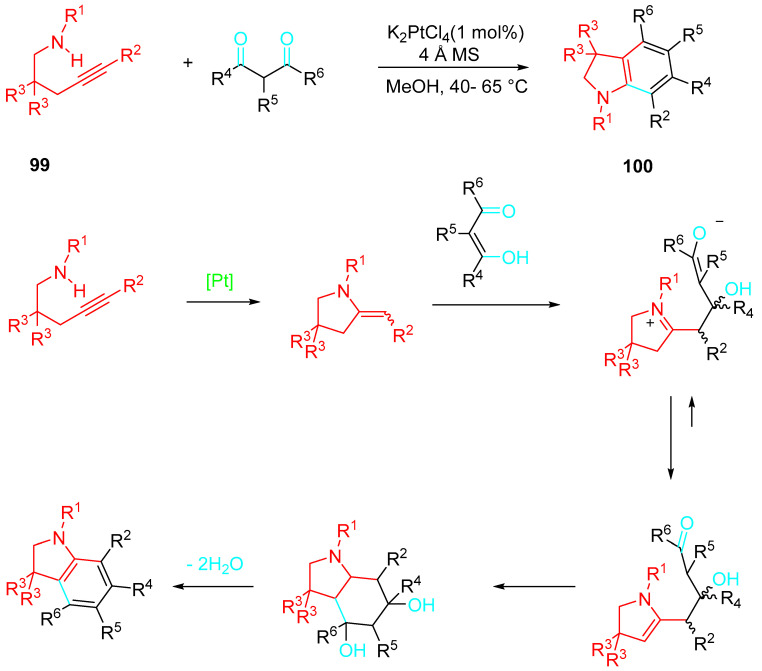
Platinum(II)-catalyzed synthesis of indolines **100**.

Although the metal-catalyzed reaction of γ-aminoalkynes **99** with 1,3-diketones is expected to afford a wide variety of products, unexpectedly the reaction accomplished the isolation of only indolines **100** with up to 99% yield. Different solvents and a variety of catalysts based on Cu, Co, Ni, Ag, Au, Pd, or Pt were screened. The results revealed that the optimized reaction conditions were observed in methanol as the solvent at 40 °C in the presence of K_2_PtCl_4_ (1 mol%) and 4Å molecular sieves. The reaction times could be shortened by subjecting the reaction to microwave irradiation. Very likely, the procedure involves a platinum-catalyzed intramolecular hydroamination of aminoalkynes to generate the corresponding enamine, which—after sequential nucleophilic attack of the enol form of the 1,3-dicarbonyl/cyclization and elimination of two water molecules—gives the indoline derivatives (Scheme 81) [96].

**Scheme 82:**
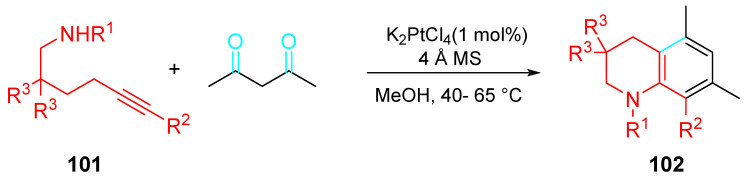
Platinum(II)-catalyzed synthesis of tetrahydroquinolines **102**.

The extension of the reaction to δ-aminoalkynes **101** gave with high yields the 1,2,3,4-tetrahydroquinolines **102** of importance in medicinal chemistry (Scheme 82).

## 5. Conclusions and Outlook

A variety of aminoalkynes can trigger sequential reactions with carbonyls to generate valuable heterocyclic scaffolds. The increasing number of aminoalkynes as building blocks has greatly widened the scope of sequential approaches to large libraries of valuable nitrogen-containing heterocyclic compounds that can be obtained from easily available reagents. Coinage metals dominated the field, and in particular, gold complexes demonstrated superior performance as catalysts for these transformations. Inexpensive and less toxic iron and zinc salts are growing in importance as efficient catalysts. As for reaction media, greener alternatives such as water, ionic liquids and solventless reactions have been reported. Advantages of microwave irradiation over conventional heating have also been highlighted. Extensive mechanistic studies allowed the identification of several intermediates and helped to explain the key role of the catalyst and the additives employed. Often, the activation of the alkyne moiety by metal catalysis is essential to boost the sequential process. We foresee that further advancements will achieve straightforward alternative easy access to a wide array of polyheterocyclic scaffolds with potentially remarkable biological activity.

## Data Availability

Not applicable.

## References

[1] Hwang E.T., Lee S. (2019). Multienzymatic cascade reactions via enzyme complex by immobilization. ACS Catal..

[2] Nicolaou K.C., Edmonds D.J., Bulger P.G. (2006). Cascade reactions in total synthesis. Angew. Chem. Int. Ed..

[3] Nicolaou K.C., Chen J.S. (2009). The art of total synthesis through cascade reactions. Chem. Soc. Rev..

[4] Oishi M., Nakanishi Y., Suzuki H. (2017). Selective incorporation of primary amines into a trizirconium imido system and catalytic cyclization of aminoalkynes. Inorg. Chem..

[5] Arcadi A., Bandini M. (2016). Gold-catalyzed synthesis of nitrogen heterocyclic compounds via hydroamination reactions. Au-Catalyzed Synthesis and Functionalization of Heterocycles.

[6] Arcadi A., Abbiati G., Rossi E. (2011). Tandem imination/annulation of γ- and δ-ketoalkynes in the presence of ammonia/amines. J. Organomet. Chem..

[7] Chang S., Lee M., Jung D.Y., Yoo E.J., Cho S.H., Han S.K. (2006). Catalytic one-pot synthesis of cyclic amidines by virtue of tandem reactions involving intramolecular hydroamination under mild conditions. J. Am. Chem. Soc..

[8] Liu X.-Y., Che C.-M. (2008). A highly efficient and selective AuI-catalyzed tandem synthesis of diversely substituted pyrrolo[1,2-*a*]quinolines in aqueous media. Angew. Chem. Int. Ed..

[9] Zhou Y., Feng E., Liu G., Ye D., Li J., Jiang H., Liu H. (2009). Gold-catalyzed one-pot cascade construction of highly functionalized pyrrolo[1,2-*a*]quinolin-1(2H)-ones. J. Org. Chem..

[10] Ma C.-L., Zhao J.-H., Yang Y., Zhang M.-K., Shen C., Sheng R., Dong X.-W., Hu Y.-Z. (2017). A copper-catalyzed tandem cyclization reaction of aminoalkynes with alkynes for the construction of tetrahydropyrrolo[1,2-*a*]quinolines scaffold. Sci. Rep..

[11] Ma C.-L., Li X.-H., Yu X.-L., Zhu X.-L., Hu Y.-Z., Dong X.-W., Tan B., Liu X.-Y. (2016). Gold-catalyzed tandem synthesis of bioactive spiro-dipyrroloquinolines and its application in the one-step synthesis of incargranine B aglycone and seneciobipyrrolidine (I). Org. Chem. Front..

[12] Galvàn A., Calleja J., Faňanás F.J., Rodríguez F. (2015). Synthesis of pyrrolidine derivatives by a platinum/Brønsted acid relay catalytic cascade reaction. Chem. Eur. J..

[13] Huple D.B., Liu R.-S. (2015). One-pot stereocontrolled synthesis of bicyclic pyrrolidine derivatives by a platinum-Brønsted acid relay cascade reaction. ChemCatChem.

[14] Fujiwara S.-I., Shikano Y., Shin-ike T., Kambe N., Sonoda N. (2002). Stereoselective synthesis of new selenium-containing heterocycles by cyclocarbonylation of aminoalkynes with carbon monoxide and selenium. J. Org. Chem..

[15] Hu Y., Huang H. (2017). Highly selective construction of medium-sized lactams by palladium-catalyzed intramolecular hydroaminocarbonylation of aminoalkynes. Org. Lett..

[16] Li X., Wang S., Wang H., Wang W., Liu L., Chang W., Li J. (2020). Synthesis of eight-membered nitrogen heterocycles via a heterogeneous PtI_2_-catalyzed cascade cycloaddition reaction of δ-aminoalkynes with electron-deficient alkynes. Adv. Synth. Catal..

[17] Li X., Jiang C., Wang X., Ren J., Zeng T., Xu X., Li J., Liu L. (2021). Platinum iodide-catalyzed formal three-component cascade cycloaddition reactions between γ-aminoalkynes and electron-deficient alkynes. J. Org. Chem..

[18] Abbiati G., Arcadi A., Bianchi G., Di Giuseppe S., Marinelli F., Rossi E. (2003). Sequential amination/annulation/aromatization reaction of carbonyl compounds and propargylamine: A new one-pot approach to functionalized pyridines. J. Org. Chem..

[19] Yang D., Liu H., Wang D.-L., Lu Y., Zhao X.-L., Liu Y. (2016). Au complex containing phosphino and imidazolyl moieties as a bifunctional catalyst for one-pot synthesis of pyridine derivatives. J. Mol. Catal. A Chem..

[20] Sotnik S.O., Subota A.I., Kliuchynskyi A.Y., Yehorov D.V., Lytvynenko A.S., Rozhenko A.B., Kolotilov S.V., Ryabukhin S.V., Volochnyuk D.M. (2021). Cu-catalyzed pyridine synthesis via oxidative annulation of cyclic ketones with propargylamine. J. Org. Chem..

[21] Savić M.P., Ajduković J.J., Plavša J.J., Bekić S.S., Ćelić A.S., Klisurić O.R., Jakimov D.S., Petri E.T., Djurendić E.A. (2018). Evaluation of A-ring fused pyridine D-modified androstane derivatives for antiproliferative and aldo-keto reductase 1C3 inhibitory activity. Med. Chem. Commun..

[22] Yan J.-Z., Li J., Rao G.-W. (2007). One-pot synthesis of new A-ring fused steroidal pyridines. Steroids.

[23] Laavola M., Haavikko R., Hämäläinen M., Leppänen T., Nieminen R., Alakurtti S., Moreira V.M., Yli-Kauhaluoma J., Moilanen E. (2016). Betulin derivatives effectively suppress inflammation in vitro and in vivo. J. Nat. Prod..

[24] Haavikko R., Nasereddin A., Sacerdoti-Sierra N., Kopelyanskiy D., Alakurtti S., Tikka M., Jaffe C.L., Yli-Kauhaluoma J. (2014). Heterocycle-fused lupane triterpenoids inhibit Leishmania donovani amastigotes. Med. Chem. Commun..

[25] Hodoň J., Frydrych I., Trhlíková Z., Pokorný J., Borková L., Benická S., Vlk M., Lišková B., Kubíčková A., Medvedíková M. (2022). Triterpenoid pyrazines and pyridines—Synthesis, cytotoxicity, mechanism of action, preparation of prodrugs. Eur. J. Med. Chem..

[26] Rabe S., Moschner J., Bantzi M., Heretsch P., Giannis A. (2014). C-H-Functionalization logic guides the synthesis of a carbacyclopamine analog. Beilstein J. Org. Chem..

[27] Simcere Pharmaceutical Group (2018). Compound as Potassium Channel Modulating Agents.

[28] Suzhou Yunxuan Pharmaceutical Co Ltd (2016). Heterocyclic Compound with Wnt Signal Path Inhibitory Activity and Application Thereof.

[29] Zhang X., Zheng J., Ma H. (2019). Heteroaryl Compounds as cxcr4 Inhibitors, Composition and Method Using the Same. Patent.

[30] Wortmann L., Lindenthal B., Muhn P., Walter A., Nubbemeyer R., Heldmann D., Sobek L., Morandi F., Schrey A.K., Moosmayer D. (2019). Discovery of BAY-298 and BAY-899: Tetrahydro-1,6-naphthyridine-based, potent, and selective antagonists of the luteinizing hormone receptor which reduce sex hormone levels in vivo. J. Med. Chem..

[31] Luo G., Chen L., Conway C.M., Denton R., Keavy D., Gulianello M., Huang Y., Kostich W., Lentz K.A., Mercer S.E. (2012). Discovery of BMS-846372, a potent and orally active human CGRP receptor antagonist for the treatment of migraine. ACS Med. Chem. Lett..

[32] C.H. Boehringer Sohn AG & Co. KG (2017). Aryl- and Heroarylcarbonyl Derivatives of Benzomorphanes and Related Scaffolds, Medicaments Containing Such Compounds and Their Use.

[33] Lowe R.A., Taylor D., Chibale K., Nelson A., Marsden S.P. (2020). Synthesis and evaluation of the performance of a small molecule library based on diverse tropane-related scaffolds. Biorg. Med. Chem..

[34] Eckhardt M., Peters S., Nar H., Himmelsbach F., Zhuang L. (2011). Boehringer Ingheleim Int, Aryl-and Heteroarylcarbonyl Derivatives of Hexahydroindenopyridine and Octahydrobenzoquinoline. U.S. Patent.

[35] Park N.Y., Lee H.S., Piao L.H., Park S.Y., Jeong W. (2022). Transition Metal Compound for Olefin Polymerization Catalyst, and Olefin Polymerization Catalyst Including Same. U.S. Patent.

[36] Faňanás F.J., Arto T., Mendoza A., Rodríguez F. (2011). Synthesis of 2,5-dihydropyridine derivatives by gold-catalyzed reactions of β-ketoesters and propargylamines. Org. Lett..

[37] Lee S., Yoo H., Park S., Yoon R., Kim S. (2023). Facile one-pot synthesis of polysubstituted pyridinium salts by annulation of enamines with alkynes. Chem. Eur. J..

[38] Changchun Haipurunsi Tech Co Ltd (2018). A Kind of Miscellaneous Anthracene Derivant and Preparation Method Thereof and Organic Luminescent Device.

[39] University Zhejlang Technology (2021). Method for Synthesizing Substituted Dihydrophenanthroline Compound.

[40] Watkins E.B., Uredi D., Motati D.R. (2020). Novel Methods for Preparation of Substituted Pyridines and Related Novel Compounds. U.S. Patent.

[41] Uredi D., Motati D.R., Watkins E.B. (2019). A simple, tandem approach to the construction of pyridine derivatives under metal-free conditions: A one-step synthesis of the monoterpene natural product, (-)-actinidine. Chem. Commun..

[42] Zhao Z., Wei H., Xiao K., Cheng B., Zhai H., Li Y. (2019). Facile synthesis of pyridines from propargyl amines: Concise total synthesis of suaveoline alkaloids. Angew. Chem. Int. Ed..

[43] Wei H., Li Y. (2019). Quick access to pyridines through 6π-3-azatriene electrocyclization: Concise total synthesis of suaveoline alkaloids. Synlett.

[44] Uredi D., Motati D.R., Watkins E.B. (2018). A unified strategy for the synthesis of β-carbolines, γ-carbolines, and other fused azaheteroaromatics under mild, metal-free conditions. Org. Lett..

[45] Uredi D., Burra A.G., Watkins E.B. (2021). Rapid access to 3-substituted pyridines and carbolines via a domino, copper-free, palladium-catalyzed Sonogashira cross-coupling/6π-aza cyclization sequence. J. Org. Chem..

[46] Lanzhou University (2018). A Kind of Polysubstituted Pyridine Derivative and Preparation Method Thereof.

[47] Chikayuki Y., Miyashige T., Yonekawa S., Kirita A., Matsuo N., Teramoto H., Sasaki S., Higashiyama K., Yamauchi T. (2020). Transition-metal-free synthesis of pyridine derivatives by thermal cyclization of *N*-propargyl enamines. Synthesis.

[48] Keskin S., Balci M. (2015). Intramolecular heterocyclization of O-propargylated aromatic hydroxyaldehydes as an expedient route to substituted chromenopyridines under metal-free conditions. Org. Lett..

[49] Hoplamaz E., Keskin S., Balci M. (2017). Regioselective synthesis of benzo[*h*][1,6]-naphthyridines and chromenopyrazinones through alkyne cyclization. Eur. J. Org. Chem..

[50] Alcaide B., Almendros P., Alonso J.M., Fernández I., Gómez-Campillosa G., Torres M.R. (2014). A gold-catalysed imine-propargylamine cascade sequence: Synthesis of 3-substituted-2,5-dimethylpyrazines and the reaction mechanism. Chem. Commun..

[51] Nie Q., Yao F., Yi F., Cai M. (2017). A heterogeneous gold(I)-catalyzed cascade annulation of aldehydes with propargylamine leading to 3-substituted 2,5-dimethylpyrazines. J. Organomet. Chem..

[52] Donald J.R., Martin S.F. (2011). Synthesis and diversification of 1,2,3-triazole-fused 1,4-benzodiazepine scaffolds. Org. Lett..

[53] Donald J.R., Wood R.R., Martin S.F. (2012). Application of a sequential multicomponent assembly process/Huisgen cycloaddition strategy to the preparation of libraries of 1,2,3-triazole-fused 1,4-benzodiazepines. ACS Comb. Sci..

[54] Nguyen H.H., Palazzo T.A., Kurth M.-J. (2013). Facile one-pot assembly of imidazotriazolobenzodiazepines via indium(III)-catalyzed multicomponent reactions. Org. Lett..

[55] Festa A.A., Raspertov P.V., Voskressensky L.G. (2022). 2-(Alkynyl)anilines and derivatives-versatile reagents for heterocyclic synthesis. Adv. Synth. Catal..

[56] Vavsari V.F., Nikbakht A., Balalaie S. (2022). Annulation of 2-alkynylanilines: The versatile chemical compounds. Asian J. Org. Chem..

[57] Kamble O.S., Khatravath M., Dandela R. (2021). Applications of ethynylanilines as substrates for construction of indoles and indole-substituted derivatives. ChemistrySelect.

[58] Murai K., Hayashi S., Takaichi N., Kita Y., Fujioka H. (2009). Tandem β-enamino ester formation and cyclization with *o*-alkynyl anilines catalyzed by InBr_3_: Efficient synthesis of-β-(*N*-indolyl)-α,β-unsaturated esters. J. Org. Chem..

[59] Arcadi A., Alfonsi M., Bianchi G., D’Anniballe G., Marinelli F. (2006). Gold-catalysed direct couplings of indoles and pyrroles with 1,3-dicarbonyl compounds. Adv. Synth. Catal..

[60] Peng C., Wang Y., Liu L., Wang H., Zhao J., Zhu Q. (2010). *p*-Toluenesulfonic acid promoted annulation of 2-alkynylanilines with activated ketones: Efficient synthesis of 4-alkyl-2,3-disubstituted quinolines. Eur. J. Org. Chem..

[61] Sivaraman M., Perumal P.T. (2014). Synthesis of 4-methyl-2,3-disubstituted quinoline scaffolds via environmentally benign Fe(III) catalysed sequential condensation, cyclization and aromatization of 1,3-diketone and 2-ethynylaniline. RSC Adv..

[62] Ortiz-Cervantes C., Flores-Alamo M., García J.J. (2016). Synthesis of pyrrolidones and quinolines from the known biomass feedstock levulinic acid and amines. Tetrahedron Lett..

[63] Wang G., Jia J., Liu G., Yu M., Chu X., Liu X., Zhao X. (2021). Copper(I)-catalyzed tandem synthesis of 2-acylquinolines from 2-ethynylanilines and glyoxals. Chem. Commun..

[64] Sakai N., Tamura K., Shimamura K., Ikeda R., Konakahara T. (2012). Copper-catalyzed [5+1] annulation of 2-ethynylanilines with an *N*,*O*-*a*cetal leading to construction of quinoline derivatives. Org. Lett..

[65] Wang Y., Peng C., Liu L., Zhao J., Su L., Zhu Q. (2009). Sulfuric acid promoted condensation cyclization of 2-(2-(trimethylsilyl)ethynyl)anilines with arylaldehydes in alcoholic solvents: An efficient one-pot synthesis of 4-alkoxy-2-arylquinolines. Tetrahedron Lett..

[66] Majumdar K.C., Taher A., Ponra S. (2010). Unusual product from condensative cyclization: Pyrano[3,2-*f*]quinolin-3,10-diones from 6-amino-5-[(trimethylsilyl)ethynyl]-2*H*-chromen-2-one and aryl aldehydes. Synlett.

[67] Ock S.K., Youn S.W. (2010). Synergistic effect of Pd(II) and acid catalysts on tandem annulation reaction for the regioselective synthesis of ring-fused quinolines. Bull. Korean Chem. Soc..

[68] Zhu C., Ma S. (2014). Sc(OTf)_3_-catalyzed bicyclization of o-alkynylanilines with aldehydes: Ring-fused 1,2-dihydroquinolines. Angew. Chem. Int. Ed..

[69] Fu W., Xu C., Zou G., Hong D., Deng D., Wang Z., Ji B. (2009). AuCl_3_-catalyzed tandem reaction of *N*-(*o*-alkynylphenyl)imines: A modular entry to polycyclic frameworks containing an indole unit. Synlett.

[70] Halim R., Scammells P.J., Flynn B.L. (2008). Alternating iodonium-mediated reaction cascades giving indole- and quinoline-containing polycycles. Org. Lett..

[71] Halim R., Aurelio L., Scammells P.J., Flynn B.L. (2013). Scaffold-divergent synthesis of ring-fused indoles, quinolines, and quinolones via iodonium-induced reaction cascades. J. Org. Chem..

[72] Kusama H., Takaya J., Iwasawa N. (2002). A facile method for the synthesis of polycyclic indole derivatives: The generation and reaction of tungsten-containing azomethine ylides. J. Am. Chem Soc..

[73] Kayeta A., Singh V.K. (2017). A one-pot synthesis of 2,2’-disubstituted diindolylmethanes (DIMs) via a sequential Sonogashira coupling and cycloisomerization/C3-functionalization of 2-iodoanilines. Org. Biomol. Chem..

[74] Subba Reddy B.V., Swain M., Madhusudana Reddy S., Yadav J.S., Sridhar B. (2012). Gold-catalyzed domino cycloisomerization/Pictet-Spengler reaction of 2-(4-aminobut-1-yn-1-yl)anilines with aldehydes: Synthesis of tetrahydropyrido[4,3-*b*]indole scaffolds. J. Org. Chem..

[75] Han X., Lu X. (2010). Cationic Pd(II)-catalyzed tandem reaction of 2-arylethynylanilines and aldehydes: An efficient synthesis of substituted 3-hydroxymethyl Indoles. Org. Lett..

[76] Yuan S., Zhang J., Zhang D., Wei D., Zuo J., Song J., Yu B., Liu H.-M. (2021). Cu(OTf)_2_-catalyzed intramolecular radical cascade reactions for the diversity-oriented synthesis of quinoline-annulated polyheterocyclic frameworks. Org. Lett..

[77] Dhandabani G.K., Mutra M.R., Wang J.-J. (2018). Palladium-catalyzed regioselective synthesis of 1-benzoazepine carbonitriles from o-alkynylanilines via 7-endo-dig annulation and cyanation. Adv. Synth. Catal..

[78] Marsicano V., Arcadi A., Chiarini M., Fabrizi G., Goggiamani A., Iazzetti A. (2021). Synthesis of 2,2,3-substituted-2,3-dihydroquinolin-4(*1H*)-ones vs. quinoline or *N*-alkenylindole derivatives through sequential reactions of 2-alkynylanilines with ketones. Org. Biomol. Chem..

[79] Dhandabani G.K., Shih C.-L., Wang J.-J. (2020). Acid-promoted intramolecular decarbonylative coupling reactions of unstrained ketones: A modular approach to synthesis of acridines and diaryl ketones. Org. Lett..

[80] Punjajom K., Ruengsangtongkul S., Tummatorn J., Paiboonsombat P., Ruchirawat S., Thongsornkleeb C. (2023). Mn(OAc)_3_-mediated one-pot condensation-oxidative annulation of 2-alkynylanilines and 1,3-ketoesters: Synthesis of 2-substituted quinolines. J. Org. Chem..

[81] Marsicano V., Chiarini M., Marinelli F., Arcadi A. (2019). Synthesis of polycyclic quinolines by means of Brønsted acid mediated reaction of β-(2-aminophenyl)-α,β-ynones with ketones. Adv. Synth. Catal..

[82] Gogoi S., Shekarrao K., Duarah A., Bora T.C., Gogoi S., Boruah R.C. (2012). A microwave promoted solvent-free approach to steroidal quinolines and their in vitro evaluation for antimicrobial activities. Steroids.

[83] Marsicano V., Arcadi A., Chiarini M., Fabrizi G., Goggiamani A., Iazzetti A. (2021). Sequential condensation/biannulation reactions of β-(2-aminophenyl)-α,β-ynones with1,3-dicarbonyls. Org. Biomol. Chem..

[84] Jash M., Das B., Chowdhury C. (2016). One-pot access to benzo[*a*]carbazoles via palladium(II)-catalyzed hetero- and carboannulations. J. Org. Chem..

[85] Abbiati G., Arcadi A., Chiarini M., Marinelli F., Pietropaolo E., Rossi E. (2012). An alternative one-pot gold-catalyzed approach to the assembly of 11H-indolo-[3,2-c]quinolines. Org. Biomol. Chem..

[86] Arcadi A., Chiarini M., D’Anniballe G., Marinelli F., Pietropaolo E. (2014). Brønsted acid catalyzed cascade reactions of 2-[(2-aminophenyl)ethynyl]phenylamine derivatives with aldehydes: A new approach to the synthesis of 2,2′-disubstituted *1H*,*1′H*-3,3′-biindoles. Org. Lett..

[87] Qian X., Gao H.-H., Zhu Y.-Z., Lu L., Zheng J.-Y. (2015). Biindole-based double D-π-A branched organic dyes for efficient dye-sensitized solar cells. RSC Adv..

[88] Brambilla E., Gritti A., Pirovano V., Arcadi A., Germani R., Tiecco M., Abbiati G. (2023). Acidic deep eutectics solvents as active media for a sustainable synthesis of biindoles starting from 2,2’-diaminotolanes and aldehydes. Eur. J. Org. Chem..

[89] Subba Reddy B.V., Swain M., Madhusudana Reddy S., Yadav J.S., Sridhar B. (2014). Gold-catalyzed 5-*endo*-*dig* cyclization of 2-[(2-aminophenyl)-ethynyl]phenylamine with ketones for the synthesis of spiroindolone and indolo[3,2-*c*]quinolone scaffolds. Eur. J. Org. Chem..

[90] Yanada R., Hashimoto K., Tokizane R., Miwa Y., Minami H., Yanada K., Ishikura M., Takemoto Y. (2008). Indium(III)-catalyzed tandem reaction with alkynylbenzaldehydes and alkynylanilines to heteroaromatic compounds. J. Org. Chem..

[91] Bouma M.J., Masson G., Zhu J. (2012). Exploiting the divergent reactivity of isocyanoacetates: One-pot three-component synthesis of functionalized angular furoquinolines. Eur. J. Org. Chem..

[92] Fayol A., Zhu J. (2004). Synthesis of polysubstituted 4,5,6,7-tetrahydrofuro[2,3-*c*]pyridines by a novel multicomponent reaction. Org. Lett..

[93] Zhao S., He Y., Gao F., Wei Y., Zhang J., Chen M., Gao Y., Zhang Y., Liu J.-Y., Guo Z. (2023). Rapid access to C2-quaternary 3-methyleneindolines via base-mediated post-Ugi Conia-ene cyclization. Chem. Commun..

[94] Borthakur U., Borah M., Deka M.J., Saikia A.K. (2016). Synthesis of tetrahydro-*1H*-indeno[1,2-*b*]pyridine via cascade cyclization and Friedel-Crafts reaction. J. Org. Chem..

[95] Hu Y., Jia Y., Tuo Z., Zhou W. (2023). Rhodium(III)-catalyzed intramolecular annulation and aromatization for the synthesis of pyrrolo[1,2-*a*]quinolines. Org. Lett..

[96] Liu X.-Y., Che C.-M. (2009). Highly efficient and regioselective platinum(II)-catalyzed tandem synthesis of multiply substituted indolines and tetrahydroquinolines. Angew. Chem. Int. Ed..

